# Those That Remain Caught in the “Organic Matter Trap”: Sorption/Desorption Study for Levelling the Fate of Selected Neonicotinoids

**DOI:** 10.3390/ijms25115700

**Published:** 2024-05-23

**Authors:** Gordana Sinčić Modrić, Jelena Marinić, Romano Karleuša, Igor Dubrović, Przemysław Kosobucki, Dalibor Broznić

**Affiliations:** 1 Department of Environmental Health, Teaching Institute of Public Health of Primorje-Gorski Kotar County, Krešimirova 52a, 51000 Rijeka, Croatia; gordana.modric@zzjzpgz.hr (G.S.M.); igor.dubrovic@zzjzpgz.hr (I.D.); 2 Department for Medical Chemistry, Biochemistry and Clinical Chemistry, Faculty of Medicine, University of Rijeka, Brace Branchetta 20, 51000 Rijeka, Croatia; jelena.marinic@uniri.hr (J.M.); romano.karleusa@uniri.hr (R.K.); 3 Department of Food Analysis and Environmental Protection, Faculty of Chemical Technology and Engineering, University of Science and Technology of Bydgoszcz, 3 Seminaryjna Street, 85-326 Bydgoszcz, Poland; p.kosobucki@pbs.edu.pl

**Keywords:** acetamiprid, imidacloprid, thiacloprid, sorption/desorption, soil, organic matter trapping

## Abstract

With projections suggesting an increase in the global use of neonicotinoids, contemporary farmers can get caught on the “pesticide treadmill”, thus creating ecosystem side effects. The aim of this study was to investigate the sorption/desorption behavior of acetamiprid, imidacloprid, and thiacloprid that controls their availability to other fate-determining processes and thus could be useful in leveling the risk these insecticides or their structural analogues pose to the environment, animals, and human health. Sorption/desorption isotherms in four soils with different organic matter (OC) content were modelled by nonlinear equilibrium models: Freundlich’s, Langmuir’s, and Temkin’s. Sorption/desorption parameters obtained by Freundlich’s model were correlated to soil physico-chemical characteristics. Even though the OC content had the dominant role in the sorption of the three insecticides, the role of its nature as well as the chemical structure of neonicotinoids cannot be discarded. Insecticides sorbed in the glassy OC phase will be poorly available unlike those in the rubbery regions. Imidacloprid will fill the sorption sites equally in the rubbery and glassy phases irrespective of its concentration. The sorption of thiacloprid at low concentrations and acetamiprid at high concentrations is controlled by hydrophilic aromatic structures, “trapping” the insecticides in the pores of the glassy phase of OC.

## 1. Introduction

Despite the important role of the Green Revolution in the sustainability of the world’s food crop production, the introduction of high-yielding, disease-resistant crop varieties raised by using agrochemicals and synthetic fertilizers in combination with modern irrigation practices depleted soil nutrients and water resources, reduced biodiversity, and increased greenhouse gas emissions [1]. These concerns call for approaches that recognize sustainable practices to food production to keep the environment safe whilst meeting the demand for food of an ever-growing population.

In the European Union (EU), polices dictated by the goals of the European Green Deal [2], the Biodiversity Strategy [3], and The common agricultural policy: 2023–2027 [4] aim to support sustainable agricultural practices and a shift to regenerative agriculture that has the potential to provide economic and social benefits, while rejuvenating the soil, fostering biodiversity, and fighting climate change. One of the stepping-stones in achieving the set goals is to reduce the use and risk of chemical pesticides by 50% by 2030.

Five synthetic neonicotinoids, including *N*-cyanoamidines (thiacloprid and acetamiprid) and *N*-nitroguanidines (imidacloprid, thiamethoxam, and clothianidin) (Table 1) [5], have become the most used insecticides in global agriculture and non-agricultural practice, with a market share of 25% in 2014 [6]. Based on information available until 2017 in Croatia, the four neonicotinoids acetamiprid, thiacloprid, imidacloprid, and thiamethoxam were applied to 91.17% of the area under agricultural management, with the majority used in fruit orchards and olive groves [7]. In agriculture, horticulture, tree nurseries, and forestry, neonicotinoids are applied as foliage sprays, soil drench, injections into irrigation water and trees, or prophylactically as seed-dressings [8], providing long-lasting protection against economically important pests, such as chewing and sucking insects [5,9,10]. The low molecular weight of neonicotinoid compounds, high water solubility, and low partition coefficient of octanol/water (log *K*_OW_) contribute to their excellent systematic activity compared to other insecticides (Table 1) [11,12]. After application, neonicotinoid insecticides are translocated to all parts of the plants body, including pollen, nectar, and plant food products [13,14], where they can affect target pests.

All five commonly used neonicotinoids belong to a class of neuro-active compounds that share the same mode of action, acting as agonists for excitatory nicotinic acetylcholine receptors (*n*AChRs), a family of ligand-gated ion channels in the central nervous system (CNS) of both vertebrates and invertebrates [15]. Their strong binding to *n*AChRs leads to rapid neurotransmission that results in paralysis and eventually death, affecting not only agricultural plant invading insects but also non-target terrestrial and aquatic invertebrate taxa and species depending on these invertebrate taxa as a food source [16,17,18]. Many of these are beneficial organisms, crucial to natural ecosystem functions, like earthworms [16] and insect pollinators, especially honeybees [17,18]. Despite knowledge gaps, evidence exists that long-term exposure to neonicotinoids leads to increasing resistance of some insect pests [19] and can adversely affect the structure, composition, diversity, and functional capacity of soil bacteria, fungi, and archaea communities [20]. In terms of the risk posed to humans, neonicotinoid (acetamiprid, clothianidin, imidacloprid, thiacloprid, thiamethoxam) residues have been frequently detected in commonly consumed food commodities, with, usually, more than one neonicotinoid present in the same food item [21,22,23,24], and their residues cannot not be eliminated through washing and peeling [25]. Findings establishing neurotoxic effects for both imidacloprid and acetamiprid in neonatal rats [26] prompted the European Food Safety Agency (EFSA) to label neonicotinoids as potential developmental neurotoxicants [27].

Out of the five most used neonicotinoids labeled as toxic to bees, acetamiprid is considered as having low toxicity [28,29]. With an LD_50_ (by contact) one hundred times higher than the one reported for thiamethoxam [30], acetamiprid is approved for use in the EU until 28 February 2033 [29]. In 2018, the European Commission prohibited outdoor uses of clothianidin, imidacloprid, and thiamethoxam, except for applications in permanent greenhouses [31,32,33], and the approval of thiacloprid was rejected in 2020 [33]. Although most neonicotinoids are banned in the EU, these substances are currently registered for use on >140 different crops in over 120 countries worldwide [34] and are projected to increase in global agriculture with the design of alternatives that exploit similar modes of action. Modern farmers can thus get caught on the “pesticide treadmill”, being forced to continuously and in increasing concentrations apply neonicotinoids with augmented toxicities [35]. Assessing both wildlife and human exposure risk from neonicotinoids requires comprehensive data collection about their fate, behavior, and ecotoxicological effects, but these data are either insufficient or of insufficient quality [30,36].

The soil is the principal inventory of neonicotinoids, playing a vital role in the distribution and fate of contamination. Only a proportion of active neonicotinoid substance applied is taken up by a plant, and in variable amounts (from 1.6 to 20%) [14], leaving residues that may undergo migration in soil (to other environmental media or non-target organisms), degradation (abiotic or biotic), or sorption/desorption processes, in which insecticide molecules become associated with the soil solid phase and retained in the soil matrix [37]. In fact, neonicotinoids are frequently detected in surface and groundwater across the world at average concentrations in surface water of tens to hundreds ng/L [10,36,37,38], and some of them occur in a variety of surface water at concentrations above the EU environmental quality standards of 0.1 μg/L [39]. Moreover, neonicotinoids are not readily biodegradable by soil microbial activity [38,40] and can persist in soils for months to years [41]. This is reflected in their high value of DT_50_ (Table 1) and their accumulation in soils after repeat application [11,12].

By partitioning neonicotinoids between the solid and the liquid phases of the soil, sorption/desorption processes are a decisive factor in determining the fate of pesticides in soil, controlling their availability for other process: sorption by the plant, migration, or degradation [9,11,40]. In turn, these processes are affected by the physical and chemical properties of the pesticides and soil [42,43,44,45]. Typically used in fate and pesticide transport mathematical models, the distribution coefficient *K*_d_ describes the efficacy of sorption and represents the ratio of the amount of pesticide sorbed to that in soil solution. *K*_d_ values, or their values normalized to the organic carbon content, i.e., *K*_OC_, predict soil sorption efficiency at lower pesticide concentrations and relate linearly the sorbed concentration of the pesticide to its concentration in solution. At high surface loadings, sorption typically becomes nonlinear, warranting the use of sorption isotherms, which relate the sorbed concentration of the pesticide to any concentration in solution. When isotherms are described by a Freundlich equation, the efficiency of sorption is characterized by the parameter *K_F_* [46,47]. Along with mechanistic considerations, the use of a Freundlich equation allows for the estimation of the sorption intensity (1/n), and the equation is applicable to non-ideal sorption on a heterogenous surface, composed of multiple surface and pore types [47]. The results in the literature report both sorption linearity and nonlinearity for acetamiprid, imidacloprid, and thiacloprid in diverse soil types [20,44,45,48,49,50,51]. According to these values, thiacloprid is the most sorbed neonicotinoid in soil, while the efficiency of sorption is similar for imidacloprid and acetamiprid. The differences in the Freundlich values of 1/n indicate the greatest heterogeneity of sorption site energies for imidacloprid and the lowest for thiacloprid. The value of the free Gibbs energy (ΔG) calculated for all three neonicotinoids by Li et al. [52] ranged from −14.6 to −19.5 kJ/mol at 21 (±2) °C, suggesting that the sorption occurs through van der Waals force, resulting in a weak and reversible sorption process.

The availability of pesticides sorbed in soil may progressively decline with time, affecting the insecticides’ uptake into plants, their leaching and runoff in the soil system, and biotic/abiotic degradation, as well as their control of weeds and pests. Recently, we demonstrated the kinetic behavior differences between acetamiprid and thiacloprid, suggesting that intra-particle diffusion is a relevant process in acetamiprid sorption, while thiacloprid was likely sorbed externally, at sites closely associated with organic matter [53]. Adding to the existing knowledge, this study aims to further clarify if sorption, and to which extent, can control the availability of acetamiprid, imidacloprid, and thiacloprid to other fate-determining processes [54] in the soil environment. As formulated in the Integrated Pest Management (IPM) principles, progress towards lower pesticide use in agriculture may be based on chemical pesticides that should be as specific as possible for the target pest, properly applied for the purpose intended and only when necessary, with the least ecosystem side effects. Since agricultural application is one of the indicators of the lifecycle of pesticides [55], acetamiprid, imidacloprid, and thiacloprid’s soil sorption/desorption parameters can be useful in modeling approaches to level the risk these contemporary insecticides or their structural analogues pose to plants and plant products, the environment, and human health. For this reason, we employed sorption/desorption studies of acetamiprid, imidacloprid, and thiacloprid with three main objectives: (a) to examine their sorption/desorption behaviors in a diverse group of soils; (b) to relate their structure and molecular variations to the nature of soil organic matter; and (c) to use the model to evaluate the possible mechanism of the sorption/desorption process.

## 2. Results

### 2.1. Physico-Chemical Characteristics of Experimental Soil

In the batch sorption/desorption tests, four natural soils were used: forest soil (soil S1), lake sediment (soil S2), and two agricultural soils (soils S3 and S4), which, according to their textural characteristics, are classified as clay loams, i.e., finely textured soil. Detailed physical and chemical characteristics of the soil samples are shown in Table 2. According to the classification of the soil acidity reaction (Thun), all analyzed soil samples belong to acidic soils, except soil S4, which is weakly acidic. The highest values of soil hydrolytic acidity (HA) and cation exchange capacity (CEC) were confirmed for soil S1 (13.39 and 60.76 cmol/kg). Soil S1 has the lowest clay content (30.75%) but also the highest amount of the total organic carbon content (TOC; 2.59%), while soil S2 has the lowest TOC content (1.06%). Based on the amount of OC, all soils are classified as weakly humic (1–3%). These amounts are within the expected range for Croatian agricultural soils (1–5% OC) [56,57,58,59]. This result can be attributed to the location from which the samples were taken, i.e., the fact that soil S1 is a forest soil, which is usually richer in OC compared to common agricultural soils. The highest amount of humic and fulvic acids (C_oxHa_ and C_oxFa_) and E465/E665 ratio were found in soil S1. The H/C ratio shows an increasing trend in hydrophilicity from soil S1 (3.30) to S2 (4.64). A lower value of the H/C ratio indicates a higher amount of aromatic compounds and –C=C– bonds in other soil organic matter components. The lower the H/C ratio is, the greater the hydrophobicity tends to be. The N/C atomic ratio ranged from 0.09 to 0.13 for soil S1 and S2, respectively, while the S/C ratio in all soil samples was almost identical. High values of N/C indicate a high content of nitrogen. The ratio (N + O)/C, i.e., the polarity index indicating the polarity of humic substances, was the highest in soil S2 and lowest in S1. The E465/E665 ratio is reciprocally related to the degree of condensation of the soil organic phase, where values below 5 indicate a high degree of condensation and the dominance of aromatic compounds, while values above 5 reflect the presence of more aliphatic and low-molecular-weight compounds. The values of the E465/E665 ratio in all soils were higher than 5, indicating moderate condensation and the dominance of low-molecular-weight aliphatic compounds (fulvic acids) that are more soluble and active in a wide range of soil pH (4–9).

### 2.2. Estimation of Nonlinear Sorption/Desorption Model in Describing the Behavior of Acetamiprid, Imidacloprid, and Thiacloprid in Soil

To estimate the time needed to achieve sorption and desorption equilibrium, the sorption and desorption kinetics of acetamiprid, imidacloprid, and thiacloprid (30 mg/L) were monitored at 20 (±1) °C for a time frame of 96 h. The results of the sorption/desorption study are presented in our previous publication [53]. In our studies, the sorption kinetics showed rapid initial insecticidal sorption within the first few hours, depending on the insecticide, and the equilibrium was reached within 47 h, while the equilibrium for the desorption reactions was reached within 94 h. The sorption and desorption processes were conducted for 96 h, and this time was sufficient to reach equilibrium conditions.

To find a mathematical model that best describes the sorption/desorption processes of acetamiprid, imidacloprid, and thiacloprid in the tested soils, three nonlinear equilibrium models were used: Freundlich’s (Equation (S1)), Langmuir’s (Equation (S2)), and Temkin’s (Equation (S3)). Experimental data modelled by the Langmuir and Temkin model provided a poorer explanation of the sorption processes for all insecticides than that provided by the Freundlich model. This is evidenced by the obtained statistical parameters, in which the fitting of experimental data by the Freundlich model generated a higher *R*^2^ (from 0.9959 to 0.9999) and a low error of SRMSE and err-% values (from 0.0944 to 0.0165 and from 7.51 to 1.31 (Table 3)) compared to the Langmuir and Temkin models (Tables S1 and S2). Desorption isotherms also coincided better with the Freundlich isotherm model (*R*^2^ from 0.9949 to 0.9985, SRMSE from 0.0993 to 0.0395, and err-% from 7.90 to 3.15) than with the Langmuir (*R*^2^ from 0.8524 to 0.9206, SRMSE from 0.4506 to 0.2764, and err-% from 35.83 to 21.98) and with the Temkin model (*R*^2^ from 0.6549 to 0.7839, SRMSE from 0.5714 to 0.3155, and err-% from 45.44 to 25.09) (Table 3, Tables S1 and S2). Since the sorption/desorption data of the entire range of the analyzed neonicotinoid concentrations were well described by the Freundlich equation, this model was chosen for the description of equilibria experiments.

### 2.3. Sorption Equilibrium Study

The sorption isotherm parameters evaluated by the Freundlich model of the three neonicotinoids in four soils are shown in Table 3. Generally, *K*_F_^sor^ is taken as a measure of sorption efficiency or sorption capacity, while 1/n reflects the heterogeneity of sorption site energies and affects the shape of the sorption isotherm. Our results indicated that the sorption of neonicotinoids was dependent on the soil type but also on the properties of the applied insecticide. Depending on the soil type, the estimated *K*_F_^sor^ values were in the range from 3.56 to 11.31 [(mg/kg)/(mg/L)]^1/n^ for acetamiprid, from 5.68 to 18.61 [(mg/kg)/(mg/L)]^1/n^ for imidacloprid, and from 6.71 to 32.60 [(mg/kg)/(mg/L)]^1/n^ for thiacloprid. The sorption capacity of all analyzed neonicotinoids decreased according to the following soil order: S1 > S4 > S3 > S2. The effect of soil physico-chemical characteristics on the sorption process will be analyzed in one of the following chapters. The soil sorption capacities for individual neonicotinoids also differed from one to the other. In all analyzed soils, a trend of increasing sorption was observed in the order of acetamiprid < imidacloprid < thiacloprid.

Various values for *K*_F_ can be found in the literature depending on the physico-chemical characteristics of the soil. Thus, Pietrzak et al. [60] in Polish soils poor in OM (0.21–1.29%) and clay (0.3–1.29%) found much lower values of *K*_F_ varying between 0.33 and 1.50 [(mg/kg)/(mg/L)]^1/n^ for acetamiprid, 0.247 and 1.043 [(mg/kg)/(mg/L)]^1/n^ for imidacloprid, and 0.376 and 3.952 [(mg/kg)/(mg/L)]^1/n^ for thiacloprid [60]. Likewise, Li et al. [52] studied the sorption of neonicotinoids on agricultural soil from Tennessee (USA) (OC content—0.279%; clay content—19%) and obtained *K*_F_ values equal to 3.02, 2.96, and 4.21 [(mg/kg)/(mg/L)]^1/n^ for acetamiprid, imidacloprid, and thiacloprid, respectively. Furthermore, lower *K*_F_ values ranging from 1.01 to 3.42 [(mg/kg)/(mg/L)]^1/n^ for imidacloprid and 1.16 to 9.06 [(mg/kg)/(mg/L)]^1/n^ for thiacloprid were detected in soils from China. Likewise, Aseperi et al. [44] and Xu et al. [61] determined a weak sorption capacity of soil from the UK (0.8–12.5% OM and 21.3–23.4% clay) and China (3.61% OM and 9.2% clay) for thiacloprid (*K*_F_, 1–11.35 and 3.44 [(mg/kg)/(mg/L)]^1/n^). In Egyptian lacustrine soil, Kandil et al. [48] determined the sorption capacity for imidacloprid to be 4.04 [(mg/kg)/(mg/L)]^1/n^. However, the soil was poor in both organic matter (0.87%) and clay (11.5%) content. For the sorption of acetamiprid in Brazilian soils, *K*_F_ values between 1.01 and 8.87 [(mg/kg)/(mg/L)]^1/n^ were obtained [62]. In our previous publication [45], we presented the results of imidacloprid sorption/desorption processes in coastal soils of Croatia. The soil clay content varied from 7.02 to 62.02%, and the OM ranged from 1.06 to 4.74%, while the obtained values of soil sorption capacity for imidacloprid were from 2.92 to 5.74 [(mg/kg)/(mg/L)]^1/n^. These values are much lower compared to the present study [45].

However, the results obtained in our study are comparable to those obtained by Olivier et al. [63], studying the sorption of imidacloprid and thiacloprid in soils from the Philippines, where the amounts of OC and clay ranged from 1.32 to 4.07% and 11.7 to 46.2%, respectively. The *K*_F_ values in this study ranged from 4.0 to 12.6 [(mg/kg)/(mg/L)]^1/n^ for imidacloprid and 7.4 to 33.2 [(mg/kg)/(mg/L)]^1/n^ for thiacloprid. Dankyi et al. [49] investigated the sorption of the same neonicotinoids on cocoa-growing soils from Ghana with different OM (1.6–48%) and clay (15–42%) content, obtaining *K*_F_ values in the range of 2.98–19.80 [(mg/kg)/(mg/L)]^1/n^ for acetamiprid, 13.27–52.14 [(mg/kg)/(mg/L)]^1/n^ for imidacloprid, and 34.46–129.99 [(mg/kg)/(mg/L)]^1/n^ for thiacloprid. The values of *K*_F_ ranged from 3.7 to 7.9 [(mg/kg)/(mg/L)]^1/n^ for acetamiprid sorption in Chinese soils (1.5–4.6 OM, 16–56% clay), indicating that the insecticide has mobile potential for surface and groundwater pollution [64]. Kodešova et al. [65] studied the sorption of thiacloprid in chernozem soils from the Czech Republic, which contained 1.14 to 5.03% OM and 11.4 to 20.3% clay, and determined the sorption capacity of the soils in the range from 3.28 to 9.96 [(mg/kg)/(mg/L)]^1/n^. The results of imidacloprid sorption capacity obtained in our study are very similar to the results obtained for Minnesota soils (USA), where *K*_F_ values were in the range from 5.0 to 15.5 [(mg/kg)/(mg/L)]^1/n^. The amounts of OM and clay in the analyzed soils ranged from 1.4 to 4.1% and 22 to 35%, which is very similar to the soil characteristics used in our study [66]. In Chinese soils (OM 0.25–4.30%, clay 9.7–49.3%), Liu et al. [50] found that the imidacloprid sorption and the *K*_F_ values varied depending on the soil. They found that the *K*_F_ decreased in the same order as the amount of organic matter in the soil.

The organic carbon partition coefficient *K*_OC_ (Equation (S5)) usually expresses the hydrophobicity of the pesticide and may be used to estimate migration and predict the behavior of an organic pesticide in the environment. The highest values of the *K*_OC_ coefficient were found for thiacloprid ranging from 499.49 to 1258.10 L/kg (Table 4), while the lowest range of *K*_OC_ values was determined for acetamiprid (from 284.79 to 436.85 L/kg). Variability in the *K*_OC_ values for the soils of different types and characteristics, and even for the soils with the same content of organic matter, indicated that not only the organic matter content but also their structure, aromaticity, and polarity affected the distribution of pesticide molecules in the soil/water system [66]. According to the classification proposed by McCall [67], acetamiprid (*K*_OC_ = 150–500 L/kg) as well as imidacloprid (except in soil S1, *K*_OC_ = 719 L/kg) can be categorized as having a medium mobility, showing less tendency to be sorbed by the examined soils. Thiacloprid with *K*_OC_ values in the range from 500 to 1258 L/kg is considered a low-mobility insecticide. In soils from the Philippines, the mean value of *K*_OC_ in the amount of 336 and 842 L/kg for imidacloprid and thiacloprid was determined by Olivier et al. [63], thus establishing the medium mobility of imidacloprid, while thiacloprid was weakly mobile in the analyzed soils. A study of the neonicotinoids’ sorption on soils from Ghana confirmed that acetamiprid shows medium mobility in the soil (average *K*_OC_ ≈ 308 L/kg), imidacloprid shows low mobility (average *K*_OC_ ≈ 832 L/kg), and thiacloprid is a practically immobile insecticide (average *K*_OC_ ≈ 2640 L/kg) [49]. Similarly, Li et al. [52] determined medium mobility of acetamiprid (*K*_OC_ = 413 L/kg) and imidacloprid (*K*_OC_ = 404 L/kg) and low mobility of thiacloprid (*K*_OC_ = 413 L/kg) in soil from Tennessee (USA). Furthermore, thiacloprid was moderately sorbed on Mediterranean semiarid climate soil (1.2% OC, 11% clay) with *K*_d_ and *K*_OC_ values of 4.88 and 407 L/kg, respectively [42]. Kandil et al. [48] determined the low mobility of imidacloprid (800.63 L/kg) in lacustrine Egyptian soil, which contained 0.87% OM and 11.5% clay. However, in Minnesota soils (USA), imidacloprid has been shown to have moderate mobility (*K*_OC_ = 340 L/kg), while in Chinese soils, its mobility was moderate (*K*_OC_ = 173–243 L/kg) or substantial (*K*_OC_ = 109–118 L/kg) [50]. Analyzing the imidacloprid sorption on Croatian coastal soils, we determined its moderate mobility (*K*_OC_ = 154–274 L/kg) [45]. The mobility of acetamiprid in Brazilian soils varied depending on the soil physico-chemical characteristics, predominantly organic matter and clay content, as well as on soil depth. Thus, in Oxisol soil (OC content 0.32 to 1.56%; soil depth 0–92 cm), acetamiprid was a highly mobile insecticide (*K*_OC_ 98–125 L/kg) [62].

To investigate the mechanisms involved in the sorption process of neonicotinoids on analyzed soils, as well as to analyze the insecticide distribution between the solid and aqueous phases, the Gibbs free energy (ΔG) was determined (Equation (S6)). The ΔG values of sorption processes are listed in Table 4 and ranged from −13.77 to −14.81 kJ/mol for acetamiprid, from −13.56 to −16.03 kJ/mol for imidacloprid, and from −15.13 to −14.81 kJ/mol for thiacloprid. The greater the absolute magnitude of the ΔG value, the greater the extent to which the sorption reaction may take place. Accordingly, the sorption of all neonicotinoids was favored on soil S1 compared to the remaining soils. In the same S1 soil, the absolute value of ΔG was in the order thiacloprid > imidacloprid > acetamiprid. Within ΔG values of 0–20 kJ/mol for physisorption, the ΔG values in this study suggest that the sorption of analyzed neonicotinoids takes place via physical processes involving weak attractive forces, primarily by the dissolution-like partition of the insecticide into soil organic matter [68]. A small negative value of ΔG indicates the exothermic nature of the reaction and a spontaneous process. Comparable ΔG values ranging from −14.6 to −19.5 kJ/mol were obtained in the study of the sorption of neonicotinoids in arable soils of Tennessee by Li et al. [52], thus proving that the sorption binding of all insecticides to the soil is mainly of a physical nature. Furthermore, the low values of ΔG also indicate that sorption between the insecticide and the soil is achieved by van der Waals attractive forces. They concluded that the sorption is relatively weak and reversible, which indicates the high mobility of the insecticide in the soil. With a *K*_OC_ value of 800.63 L/kg for imidacloprid sorption on Egyptian soil, a ΔG value of −16 kJ/mol was obtained [48]. The same insecticide showed similar behavior in soils from China, where the ΔG was in the range between −11.46 and −13.61 kJ/mol, thus demonstrating that the primary mechanism of the sorption process is the dissolution of the insecticide molecule into organic matter [52]. ΔG values in the range for physisorption from −11.69 to −13.68 kJ/mol were found for imidacloprid sorption onto Croatian olive orchards’ soils [45].

The second parameter obtained by fitting sorption curves with the Freundlich model is 1/n, reflecting the energy distribution of sorption sites; it is sometimes referred to as the heterogeneity index [69]. Clearly, a perfect linearity would exhibit 1/n ≈ 1, indicating equal energies for all sites, i.e., a homogeneous surface. In our study, the 1/n values were <1 (Table 3) and ranged from 0.772 (soil S2) to 0.848 (soil S1) for acetamiprid, from 0.704 (soil S4) to 0.895 (soil S1) for imidacloprid, and from 0.665 (soil S4) to 0.829 (soil S2) for thiacloprid. These values are consistent with those reported in the literature. Contrary to our results, Aseperi et al. [44] demonstrated that as the OC amount of the soil increased, the 1/n value decreased. This behavior indicates that the initial slope of the isotherm was nonlinear with respect to the concentration in the aqueous phase. Of the three analyzed neonicotinoids, the smallest average deviation from linearity was determined for acetamiprid, 20%, while the deviations for the remaining two insecticides amounted to about 24%.

### 2.4. Desorption Equilibrium Study

The desorption Freundlich coefficient values (*K*_F_^des^) obtained for the tested soils were higher than the sorption values (*K*_F_^sor^), while the desorption 1/n values were lower than the Freundlich sorption equilibrium values (Table 3). *K*_F_^des^ values varied from 6.73 to 15.27 [(mg/kg)/(mg/L)]^1/n^ for acetamiprid, from 10.76 to 26.70 [(mg/kg)/(mg/L)]^1/n^ for imidacloprid, and from 17.26 to 64.45 [(mg/kg)/(mg/L)]^1/n^ for thiacloprid. For all neonicotinoids under study, the highest *K*_F_^des^ values were in soil S1 (clay loam soil with 2.59% OC) followed by soils S4 and S3, while soil S2 (clay loam soil with 1.06% OC) exhibited the lowest *K*_F_^des^. A higher *K*_F_^des^ value indicates a stronger affinity for the insecticides, i.e., weaker desorption.

For the second desorption parameter, 1/n, the constants ranged from 0.732 to 0.848 for acetamiprid, from 0.699 to 0.881 for imidacloprid, and from 0.640 to 0.826 for thiacloprid. The deviations from the linear function ranged from 15% (soil S1) to 27% (soil S3) for acetamiprid, from 12% (soil S1) to 30% (soil S4) for imidacloprid, and from 17% (soil S2) to 36% (soil S4) for thiacloprid. Higher *K*_F_^des^ values compared to sorption values, but still lower than those obtained in this study, were shown in the research by Zhang et al. [20], with values ranging from 1.20 to 3.76 [(mg/kg)/(mg/L)]^1/n^ and from 1.55 to 10.9 [(mg/kg)/(mg/L)]^1/n^ for imidacloprid and thiacloprid, respectively. In the same study, the values of the nonlinearity coefficient, 1/n, were in the range from 0.650 to 1.02 for imidacloprid and from 0.645 to 0.943 for thiacloprid. The values of imidacloprid desorption coefficients (*K*_F_^des^ and 1/n) for soil S2 are similar to those obtained for Egyptian soil (*K*_F_^des^ = 9.33 [(mg/kg)/(mg/L)]^1/n^ and 1/n = 0.781) [48].

As can be seen in Table 3, 1/n^sor^ > 1/n^des^, indicating that a significant amount of the sorbed neonicotinoids is difficult to desorb and that desorption cannot be predicted from sorption isotherms. To estimate the discrepancies between the sorption and desorption isotherms, hysteresis coefficients *H* and *λ* were calculated (Equations (S7) and (S8)), and these values for the tested soils are presented in Table 4. Hysteresis is related to a shift in the sorption and desorption isotherms [70]. The *H* values in all cases were <1, indicating that all neonicotinoids showed sorption/desorption hysteresis to some extent. When the value of *H* is lower, sorption/desorption hysteresis is more pronounced with higher nonlinearity, so the desorption rate is slower in relation to the sorption rate. The same trend of a decreasing value of coefficient *H* was not observed with all neonicotinoids. For acetamiprid, increased hysteresis was in the order soil S1 < soil S4 < soil S3 < soil S2, while for imidacloprid and thiacloprid, the order was soil S3 < soil S2 = soil S4 < soil S1 and soil S3 < soil S4 < soil S1 < soil S2. Kandil et al. [48] determined the occurrence of sorption/desorption hysteresis when studying the behavior of imidacloprid in Egyptian soils (*H* = 0.898). Since the value of *H* was less than 1, they assumed that imidacloprid sorption processes onto the soil were mostly irreversible in nature. The occurrence of imidacloprid sorption/desorption hysteresis was reported in the soils of the state of Minnesota (USA). The obtained hysteresis coefficient values varied depending on the soil and the insecticide concentration (0.26 to 0.64) [66]. A discrepancy between the sorption and desorption isotherms for imidacloprid in Croatian soils was found in our previous publication. For all analyzed soils, the *H* values were lower than 1, and we found that the hysteresis was more pronounced in soils with a higher content of OM, primarily humic acids [53]. Cox et al. [66] point out the important fact that soil with a stronger sorption capacity at higher imidacloprid concentrations showed higher desorption compared to other soils, which was also confirmed by a higher hysteresis coefficient. Furthermore, they observed that the slopes of the desorption isotherms, showing the intensity of desorption, are smaller at lower imidacloprid concentrations and, accordingly, the hysteresis coefficients decrease with the concentration of the solution. This indicates that the lower the imidacloprid concentration, the more difficult the desorption of the insecticide.

### 2.5. Sorption and Desorption Isotherms

**Figure 1:**
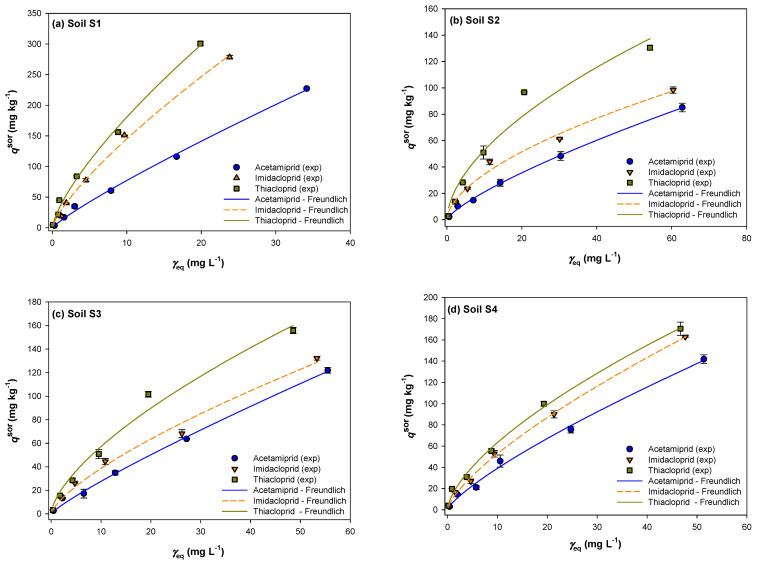
Freundlich isotherms for sorption of acetamiprid, imidacloprid, and thiacloprid in tested soils S1–S4 (**a**–**d**). Values are means ± standard deviations. Symbols and lines represent the experimental and theoretical curves represented by the Freundlich nonlinear equilibrium model, respectively.

Neonicotinoids’ sorption isotherms, represented by the mass of each insecticide sorbed by the soil [*q*_e_^sor^ (mg/kg)] vs. the insecticide equilibrium concentration [*γ*_e_ (mg/L)], are shown in Figure 1. Analyzing the dependence of the total added concentration of insecticide on the sorbed amount of insecticide, it can be inferred that the rate of sorption increases in the order of acetamiprid < imidacloprid < thiacloprid in all analyzed soils. Based on the slope coefficient of the linear function, the maximum sorption rate (*k*) was determined, which was 3.7207 for thiacloprid, 3.4276 for imidacloprid, and 2.8016 for acetamiprid. The highest sorption rate of all analyzed neonicotinoids was found in soil S1 and the lowest in soil S2 (1.0297 vs. 2.8016 for acetamiprid, 1.1608 vs. 3.4776 for imidacloprid, and 1.6300 vs. 3.7207 for thiacloprid). Furthermore, the strongest sorption capacity for all neonicotinoids was recorded in soil S1 (300.63, 278.45, and 227.16 for thiacloprid, imidacloprid, and acetamiprid, respectively) and the weakest in soil S2 (130.47, 98.32, and 85.13 for thiacloprid, imidacloprid, and acetamiprid, respectively).

In general, the sorption isotherms have a similar shape, but there are significant differences between the individual curves, indicating different sorption efficiencies and heterogeneity of the sorption sites’ energies among the tested soils, as well as among the insecticides. The shape of the sorption isotherms is nonlinear (1/n < 1), with the highest slope (d*q*_e_ = d*γ*_e_) at the initial stage of the curve and a steady decrease afterwards. The slopes of isotherms indicated that as the initial concentrations of insecticides increased, the percentage sorbed by the soil decreased. This fact is confirmed by the obtained sorption results, which indicate that soil S1 sorbed 91.8% of the initial thiacloprid concentration (1 mg/L), and the sorbed percentage decreased to 75.2 at the initial concentration of 80 mg/L. The same trend of decrease in the sorbed percentage was achieved in all analyzed soils and for all insecticides. Zhang et al. [71] found that the sorption of thiacloprid was nonlinear and highly concentration-dependent. They obtained a significant reduction in *K*_d_ values when the initial insecticide concentration in solution increased from 0.05 to 5 mg/L. All the mentioned assumptions indicate that the sorption isotherms of all analyzed neonicotinoids can be classified as an L curve according to Giles’ classification. An L curve is a common curve type for neonicotinoids’ sorption in various soils and surfaces [72]. Its shape indicates that the sorption is more efficient at a low solute concentration and becomes increasingly hindered as the number of vacant sorption sites diminishes. This in turn implies there is no cooperative sorption and that solute molecules are most likely sorbed flat [72].

**Figure 2:**
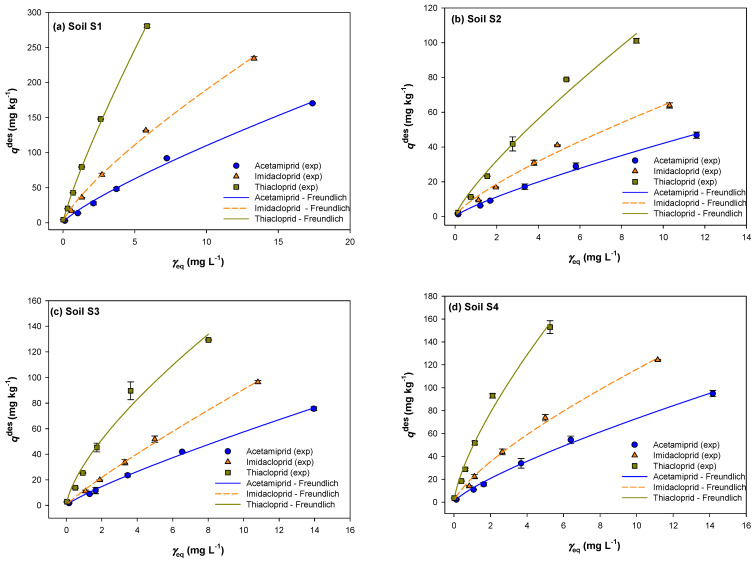
Freundlich isotherms for desorption of acetamiprid, imidacloprid, and thiacloprid in tested soils S1–S4 (**a**–**d**). Values are means ± standard deviations. Symbols and lines represent the experimental and theoretical curves represented by the Freundlich nonlinear equilibrium model, respectively.

In addition to sorption isotherms which give information about the insecticide quantity sorbed onto a soil, desorption experiments are required to study the intensity of the soil–insecticide interaction involved. The desorption isotherms of analyzed neonicotinoids for tested soils are presented in Figure 2. Desorption isotherms are shown as a function of the remaining sorbed amount of each insecticide [*q*_e_^des^ (mg/kg)] and the equilibrium desorbed concentration [*γ*_e_ (mg/L)] of the insecticide in the solution. The shape of the desorption isotherms is visually similar to the sorption isotherms at lower applied concentrations (1 and 5 mg/L), while at higher concentrations (10–80 mg/L), the shape of the isotherms varied significantly.

The desorption capacity was determined by calculating the non-desorbing amount of insecticide, which remained after the sorption process. Among neonicotinoids, the highest remaining sorbed amount after the desorption process remained in soil S1 and the smallest in soil S2. In the case of acetamiprid, this amount was in the range from 2.87 to 170.37 mg/kg, compared to the sorbed amount from 3.59 to 227.16 mg/kg for soil S1, representing 80 and 75% of the non-desorbed fraction, respectively. On the contrary, in soil S2, acetamiprid was retained in a significantly lower amount (the retained amount was in the range from 1.29 to 46.82 mg/kg), representing 62 and 55% of the initially sorbed fraction. Furthermore, in the same soil S1, imidacloprid and thiacloprid were retained in a higher percentage than acetamiprid, with values in the range from 90 to 84% and 95 to 93%, respectively. The highest non-desorbed amount recorded for thiacloprid probably results from interactions between the insecticide and soil colloid phase through chemical bonding, which is stronger and therefore more stable than with other insecticides. The highest desorbed amount of analyzed insecticide was found in soil S2 and was in the range acetamiprid (45%) > imidacloprid (35%) > thiacloprid (23%). The same behavior of the desorbing fraction was found in the other analyzed soils. Based on the linear function of the sorbed amount and the desorbed percentage (%) of insecticides, the desorption rate was determined. Acetamiprid showed the highest desorption rate in soil S2 (*k* = 0.0849), while the desorption rate of thiacloprid in soil S1 was the lowest (*k* = 0.0049). For all analyzed neonicotinoids, desorption was most pronounced at the highest initial insecticide concentration of 80 mg/L. It should be noted that in soils S2 and S3, acetamiprid desorption was significant even at lower insecticide concentrations (20 and 40 mg/L). Comparing the percentage of the desorbed amount of each insecticide with the initial insecticide sorbed concentration, an increasing trend was observed, which is most intense for acetamiprid in all analyzed soils. Contrary to our results, Nemeth-Konda et al. [51], who studied the sorption/desorption of imidacloprid in brown forest soil (1.16% OM, 15.4% clay), found that the percentage of desorbed imidacloprid decreased with increasing initial sorbed concentration.

### 2.6. Effect of Physico-Chemical Soil Characteristics on Acetamiprid, Imidacloprid, and Thiacloprid Sorption/Desorption Parameters

Statistical correlations between neonicotinoid sorption/desorption parameters estimated by the Freundlich model and the physico-chemical soil properties are presented in Table 5, Table 6 and Table 7. Correlation analyses of pooled sorption data for all insecticides indicated the significant, strong, and positive correlation of $K_{F}^{sor}$ with HA, CEC, TOC, C_oxFa_, and the ratio E465/E665 (*R*^2^ > 0.87, *p* < 0.005), while the correlation with clay content and the ratios C/H, N/C, S/C, O/C, and (N + O)/C was statistically significant but negative (*R*^2^ > −0.76, *p* < 0.028). Similar behavior as with $K_{F}^{sor}$ was achieved for the organic carbon partition coefficient (*K*_OC_ parameter) of all analyzed insecticides and soil characteristics with the difference that a statistically significant negative correlation with soil pH was achieved (*R*^2^ > −0.71, *p* < 0.047), while the dependence with the ratios H/C, O/C, and (N + O)/C was not significant. The next sorption parameter, the nonlinearity coefficient 1/n^sor^, showed a different dependence on the TOC amount compared to $K_{F}^{sor}$. Namely, a positive significant correlation of 1/n^sor^ and TOC was achieved only for acetamiprid (*R*^2^ = 0.74, *p* = 0.035). It is interesting that the 1/n^sor^ of imidacloprid showed a significant negative dependence on soil pH (*R*^2^ = −0.94, *p* = 0.001) which was not observed with the other two insecticides. Furthermore, the correlation of the acetamiprid 1/n^sor^ parameter with the ratio E465/E665 and C_oxFa_ was positive and statistically significant (*R*^2^ > 0.74, *p* < 0.035), while the remaining two insecticides did not show statistical dependence with the analyzed parameter. Only the thiacloprid 1/n^sor^ parameter was positively correlated with the ratio H/C, while the parameter for acetamiprid and imidacloprid was significantly influenced by the ratio of N/C and S/C. For all analyzed insecticides, the molar free Gibbs energy, ΔG, showed a strong, positive, and statistically significant effect with clay amount (*R*^2^ > 0.85, *p* < 0.008), while the correlation with HA, CEC, TOC, C_oxFa_, and the ratio E465/E665 was statistically significant but negative (*R*^2^ > −0.72, *p* < 0.042). The influence of the TOC on the ΔG was not significant only in the case of the acetamiprid sorption.

The desorption parameter $K_{F}^{des}$ of all insecticides was significantly and positively correlated with the ratio HA, CEC, TOC, C_oxFa_, and the ratio E465/E665 (*R*^2^ > 0.77, *p* < 0.027), while the correlations with the ratios H/C, N/C, O/C, and (N + O)/C were significant but negative *R*^2^ > −0.87, *p* < 0.004). The parameter 1/n^des^ for imidacloprid desorption showed a strong, positive, and statistically significant effect with the ratio HA, CEC, and C_oxFa_ (*R*^2^ > 0.71, *p* < 0.047), while the correlation with pH, clay, and the ratios N/C and S/C was negative (*R*^2^ > −0.87, *p* < 0.001). With the remaining two insecticides, neither of the correlations were statistically significant for the 1/n^des^ parameter. The imidacloprid hysteresis coefficient *H* was positively correlated with HA, CEC, TOC, C_oxFa_, and the ratio E465/E665 (*R*^2^ > 0.73, *p* < 0.039) and negatively correlated with pH, clay, and the ratios N/C and S/C (*R*^2^ > −0.82, *p* < 0.013). At the same time, the hysteretic coefficient *λ* showed the opposite dependence compared to the coefficient *H*. In the case of acetamiprid, the hysteretic coefficients showed a significant correlation with TOC and the ratios E465/E665, H/C, O/C, and (N + O)/C, while for thiacloprid, no significant correlations were established.

Correlation analysis indicated that multiple soil physico-chemical properties, such as the OM and clay content, CEC, and the presence and structure of humic and fulvic acids, are dominant factors that can explain the differences in the binding affinities of the analyzed neonicotinoids. Although the overall positive influence of humic acids on the sorption capacity of neonicotinoids was not observed, it should be noted that all soils except soil S3 showed stronger binding of insecticides at higher amounts of humic acids (*R*^2^ > 0.9911). Furthermore, the correlation analysis did not find a statistically significant dependence between the coefficient 1/n and the total OC, but if soil S1 is excluded from the analysis, the higher OC content causes a greater deviation from the linearity of the sorption isotherms. All these facts indicate that the neonicotinoids’ sorption/desorption behavior in soils is influenced by several physico-chemical soil characteristics, the effect of which should not be studied separately but cumulatively. For this reason, in order to determine the soil physico-chemical properties that dominantly affect the sorption/desorption parameters of neonicotinoids, principal component analysis (PCA) and multiple linear regression were performed. Although numerous studies have shown the dominant role of the OC amount in the sorption of neonicotinoids, the role of clay minerals and soil CEC cannot be discarded [49,50,52,66].

### 2.7. Determination of the Dominant Physico-Chemical Soil Characteristics on the Sorption/Desorption Processes of Acetamiprid, Imidacloprid, and Thiacloprid

**Figure 3:**
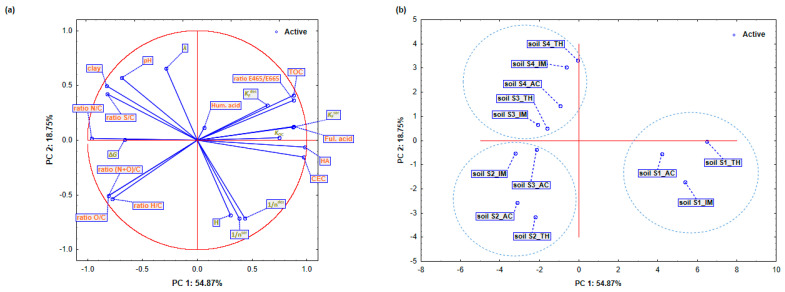
Results of principal component analysis (PCA) of physical and chemical properties of tested soils and evaluated parameters for acetamiprid, imidacloprid, and thiacloprid sorption/desorption equilibrium processes in the tested soils (S1–S4) obtained by the Freundlich nonlinear equilibrium model represented by two main components (PC 1 and PC 2). Projections of (**a**) active variables (sorption/desorption parameters of analyzed insecticides and physico-chemical parameters of the soils) and (**b**) cases (soils and insecticides) on the factor-plane.

In order to distinguish changes in the sorption/desorption behavior of the analyzed insecticides for each type of soil and to determine which of the physico-chemical characteristics has a dominant effect on the sorption and desorption of insecticides, it is extremely important to perform a global interpretation of the data. For this reason, principal component analysis (PCA) was applied, which groups physico-chemical soil characteristics and insecticide sorption/desorption parameters into clusters. Evaluated insecticide sorption/desorption parameters and soil characteristics were used as variables for the analysis, insecticides as the active case variable and soils as a group variable. The results of the PCA analysis are depicted in Figure 3 and in Table 8. Table 8 shows the first four PCs with eigenvalues greater than 1.0 which were retained in the analysis. With four main components, it is possible to explain as much as 93.98% of data variance. The first component PC1 contributes with more than half of the total variability in the amount of 54.87%, while the remaining parts of 18.75, 12.28, and 8.08% belong to PC2, PC3, and PC4.

Figure 3a,b show the projection of the active variables (soil characteristics, estimated sorption/desorption parameters of applied insecticides) and cases (soils and insecticides) in the factor-plane. It can be seen that the soils were grouped into three clusters depending on the analyzed physico-chemical characteristics of the soil: (1) the cluster of soil S1 with the highest content of TOC, fulvic acids, HA, CEC, and the ratio E465/E665 localized on the positive side of PC1, (2) the cluster of soils S3 and S4 with the highest clay amounts, pH values, and the ratios N/C and S/C occupying the positive side of PC2, and (3) the cluster of soil S2 in which the ratios O/C, H/C, and (N + O)/C dominate and is localized on the negative side of PC1. In the first cluster, soil S1 shows the strongest binding and releasing capacity of all analyzed insecticides (Figure 3a,b). In addition, it can be observed that of all the insecticides, thiacloprid shows the strongest sorption capacity in soil S1. This is supported by the fact that in the second quadrant, the positive sides of PC1 are grouped variables, $K_{F}^{sor}$, $K_{F}^{des}$, and *K*_OC_, which precisely indicate the strength of the sorption capacity. The sorption/desorption capacity of thiacloprid on soil S1 is primarily dependent on the soil characteristics localized in the positive region of PC1 (Figure 3a). The sorption of imidacloprid and acetamiprid on soil S1 is dominantly dependent on the CEC of the soil, and it was characterized with more pronounced hysteresis (*H* coefficient), indicating the strongest discrepancy between the sorption/desorption isotherm. In cluster 2 (positive site of PC2), it can be noticed that the sorption capacity of imidacloprid and thiacloprid on soil S4 is stronger than on soil S3. In the same cluster, there is soil S4 where acetamiprid sorption takes place. In the considered soils, insecticide sorption is dominantly dependent on the clay content and soil acidity, but the sorption of thiacloprid on soil S3 and acetamiprid on soil S4 was influenced by the presence of soil humic acids. Furthermore, the occurrence of hysteresis between the sorption/desorption isotherms characterized by the coefficient *λ* was observed. Finally, in cluster 3, soil S2 with sorption of all three analyzed insecticides and soil S3, where acetamiprid sorption takes place, are located. In the mentioned soils, the sorption/desorption processes are dominantly dependent on the sorption Gibbs free energy (ΔG).

The interpretation of the main components (PC1–PC4) was performed using eigenvectors (Table 8). Component PC1 was defined by the variable characterizing physico-chemical soil properties: HA, CEC, TOC, and the ratios E465/E665 and N/C. The CEC content exhibited the highest eigenvector value (0.340), while the eigenvector values of HA and the ratio N/C were within 10% of the CEC eigenvector value. Although all parameters show significant statistical dependencies (Table S3), the HA parameter was included in the calculation of the CEC parameter, leading to the selection of the CEC parameters among PC1 indicators. Among the estimated sorption/desorption parameters, only *K*_F_^sor^ defined the PC1 component (Table 8). This parameter showed a statistically significant dependence with most of the soil physico-chemical characteristics, and for this reason, it was taken as a sole indicator for assessing the sorption/desorption capacity of the analyzed insecticides. The PC2 component was defined by the ratio H/C and the Freundlich nonlinearity coefficients, 1/n^sor^ and 1/n^des^ (−0.360). Analyzing their correlation with the soil physico-chemical characteristics, it was observed that both parameters were significantly correlated with the pH values and clay content (Table S3). Therefore, both parameters were ultimately considered as indicators of the sorption/desorption soil capacity for the analyzed insecticides. In PC3, the eigenvector values were dominated by the coefficient *K*_F_^des^ (−0.406), and none of the parameters were within 10% of *K*_F_^des^. Accordingly, the parameter *K*_F_^des^ was retained as the most important indicator for PC3. The percentage of humic acids (C_oxHa_) in the PC4 component was selected as the physico-chemical characteristic describing the sorption/desorption of insecticides on the soil due to its highest eigenvector value, and no other soil characteristics were within 10% of the C_oxH_ value.

Based on the PCA and correlation analysis, the soil capacity for the sorption/desorption of neonicotinoids was screened to include the following physico-chemical characteristics: CEC, humic acids, and the H/C ratio. These parameters encompassed the physical and chemical properties of the soil. Based on the PCA results, each PC explained a certain percentage of the variations in the total data set (TDS). This percentage provided the weighting factor when the variance from each PC was divided by the cumulative variance (93.98%; Table 8), which was derived from PCs with eigenvalues greater than 1. The weighting factors for the variables in PC1 (CEC and *K*_F_^sor^), PC2 (1/n^sor^ and 1/n^des^), PC3 (*K*_F_^des^), and PC4 (humic acids) were 0.58, 0.20, 0.13, and 0.09, respectively.

Relations between the soil physico-chemical characteristics and the sorption/desorption parameters of the studied insecticides in the soil selected by PC analysis were analyzed by multiple linear regressions. Multiple linear regression determines the cumulative effect of different soil properties on the sorption/desorption parameters and leads to a linear predictive model for the sorption/desorption soil capacity for insecticides. Regression analysis between selected soil properties (CEC, Hum. acid content (C_oxHa_), ratio H/C) and sorption/desorption coefficients (*K*_F_^sor^, *K*_F_^des^, 1/n^sor^ and 1/n^des^) resulted in the relations represented in Table 9.

It is evident that the best correlation (*R*^2^ value) and the highest significance (*p* value) of the independent variables (humic acids, ratio H/C, and CEC) was obtained for the sorption of thiacloprid represented by $K_{F}^{sor}$. The *R*^2^ = 0.998 and *p* < 0.001 values indicated a good relationship between the sorption parameters and selected soil properties. Therefore, both humic acids and the H/C ratio had a dominant negative effect on the sorption parameter, while the effect of CEC was weak and positive. Positive relationships between the coefficients ${1/n}^{sor}$ and ${1/n}^{des}$ with humic acids and the H/C ratio and a positive relationship with CEC were found for imidacloprid and thiacloprid. The worst correlation (*R*^2^ = 0.642) and the lowest significance (*p* = 0.0736) were obtained between the acetamiprid ${1/n}^{sor}$ parameter and selected soil properties. The ${1/n}^{sor}$ value was negatively related to the amount of humic acids and the H/C ratio and positively related to the CEC value. The parameter $K_{F}^{des}$ for all neonicotinoids was dominantly influenced by the ratio H/C. Multiple linear regression equations suggested that the content of humic acids and their hydrophobicity or hydrophilicity predominantly influenced the insecticides’ sorption and desorption on the tested soils.

## 3. Discussion

Research conducted over the last twenty years on the behavior of neonicotinoid insecticides in soils has indicated their significant presence in the cycle of substance circulation in the environment. It is important to know their bioavailability in the soil, i.e., the fraction of insecticides that can be desorbed or degraded over time, since only the insecticide fraction that is bioavailable, but not the insecticide total amount, will pose a risk to environmental ecosystems. In general, the bioavailability of insecticides is controlled by numerous soil physico-chemical properties and by the physico-chemical interactions of insecticide molecules and soil colloids, while the conditions of the soil system, such as the pH, temperature, and humidity, play an important role in determining the direction and intensity of a specific interaction. It has been suggested that an increase in temperature significantly reduces the sorption of neonicotinoids on soil particles, but it also increases their desorption, which occurs due to an increase in the kinetic energy of molecules and a weakening of their intermolecular interactions with soil surfaces. Broznić et al. [59] found that increasing the temperature from 20 to 40 °C caused approximately two times weaker sorption and stronger desorption of imidacloprid. Changes in temperature have a significant impact on the neonicotinoid’s behavior in soils in areas with high air temperatures but also when they are applied in greenhouse conditions. Namely, higher temperatures increase the volatility and mobility of neonicotinoids and facilitate their release from soil particles into the water phase, which leads to faster migration of neonicotinoids in the environment and increases the risk of ground- and surface-water pollution. It is known that the geochemical properties of soil, such as the amount of organic matter and its properties, influence the type and intensity of interactions between insecticide molecules and the soil. For this reason, the interactions of neonicotinoids with the components of soil organic matter are a decisive factor in determining the bioavailability of these insecticides.

Xing and Pignatello, Ref. [73], hypothesized that soil organic matter consists of flexible rubbery and inflexible glassy phases. A characteristic of the glassy phase is the presence of unrelaxed free volumes in the form of internal pores of nanometer sizes. Sorption in the rubbery phase takes place by the mechanism of dissolution in the solid phase (distribution), and this process is linear and non-competitive, unlike sorption in the glassy phase, which occurs by a dual mechanism that includes both distribution and sorption in the internal pores. Sorption in the glassy phase is nonlinear and competitive. Due to its high water solubility and moderate polarizability, imidacloprid will have the strongest binding tendency to the soil organic matter’s rubbery phase (through the highly polar nitroguanidine group), but it will also be partially sorbed by the polar components of the soil glassy phase. Due to its weak hydrophobicity and high water solubility, acetamiprid will be weakly sorbed by the hydrophobic compounds of the soil glassy phase but will interact with the polar functional groups of the rubbery phase. However, its overall sorption will be weak to moderate due to the presence of less polar functional groups in the molecule. Due to its high hydrophobicity and poor water solubility, thiacloprid will have the greatest tendency for sorption on the hydrophobic parts of the glassy phase of the soil.

**Figure 4:**
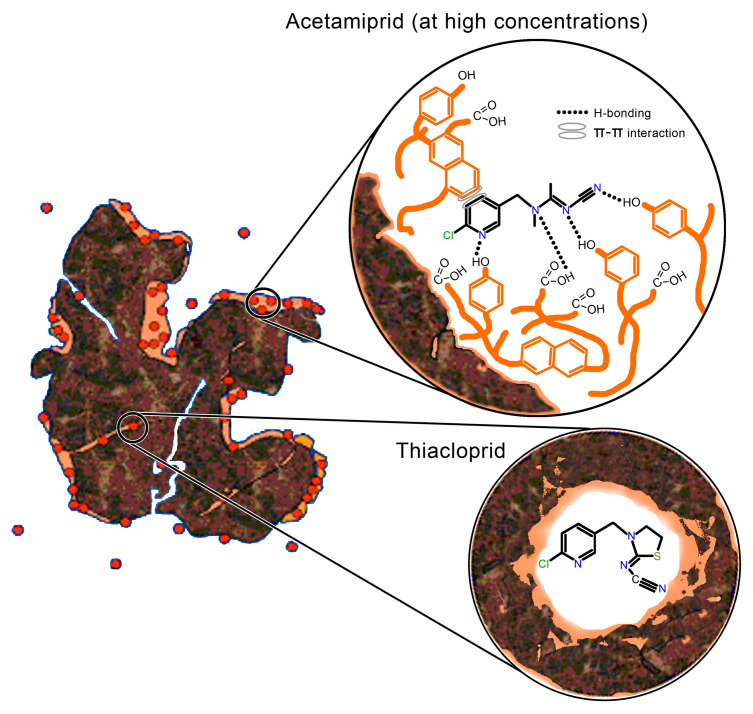
Schematic representation of possible mechanisms for acetamiprid and thiacloprid sorption.

Therefore, we propose that at low insecticide concentrations, most acetamiprid molecules will first occupy sorption sites in the rubbery aliphatic phase, while at higher concentrations, sorption sites in the hydrophilic glassy regions begin to fill. Imidacloprid will fill the sorption sites equally in the rubbery and glassy phases irrespective of its concentration. Thiacloprid, as the most hydrophobic molecule, will fill sorption sites in the glassy soil phase at low insecticide concentrations, while at higher concentrations, sorption sites in the rubbery soil phase will begin to be filled (Figure 4). Since the sorption region contains a limited number of high-energy sorption sites, neonicotinoid molecules occupy these sites first at low concentrations, which means that at low concentrations, the sorption mechanism dominates the partition [74]. Therefore, the partition and specific sorption of neonicotinoids most likely occur simultaneously. From the aspect of bioavailability, it is considered that neonicotinoid molecules sorbed in the rubbery phase will be completely bioavailable, while those sorbed in the glassy phase will be poorly bioavailable.

In addition, De Jonge and Mittelmeijer-Hazeleger [75] showed that natural organic compounds, such as humic acids, have high microporosity, with pore radius of <20 Å, so we assumed that neonicotinoid binding may be the result of the irreversible “trapping” of molecules in the pores of natural organic matter. If we assume that the pore radius is 10 Å, the calculated pore volume is about 4200 Å^3^. Since the volumes of one molecule of acetamiprid, imidacloprid, and thiacloprid are 316, 276, and 291 Å^3^, respectively [12], it is possible that “irreversible trapping” caused the trapping of molecules in the pores. However, this assumption cannot justify the difference in the sorption behavior of neonicotinoids because their molecules have approximately the same volume.

To describe the properties of organic matter and the correlation with sorption/desorption parameters, both the (N + O)/C ratio and the H/C atomic ratios were used, with the first representing the polarity index [74,76] and the latter indicating the degree of aromaticity of organic matter [10,77,78]. Glassy organic matter is less polar than rubbery matter and exhibits a greater degree of aromatic character. The calculated H/C molar ratios showed an increasing trend in hydrophilicity from soil S1 (3.30) to S2 (4.64). A lower value of the H/C ratio indicated a greater amount of aromatic compounds with –C=C– bonds in the organic phase of the soil, i.e., a higher amount of hydrophobic groups. With a higher value of the H/C ratio, a higher hydrophilicity was observed. The H/C ratio indicates the highest abundance of hydrophobic groups in soil S1, while soil S2 is the most hydrophilic. Higher O/C and (N + O)/C ratios indicated high polarity and a higher content of oxygen functional groups in the organic matter of soil S2. It can be concluded that aromaticity will generally favor sorption, while a high amount of hydrophilic carboxyl groups will suppress neonicotinoid sorption. Polar groups can participate in the formation of hydrogen bonds, which can significantly affect the three-dimensional structure of organic matter. For example, if divalent cations (e.g., Ca^2+^) are bound to functional groups, the formation of bridges between polar groups and “twisting” of macromolecules occurs, which leads to the formation of hydrophobic cavities, suggesting that under such conditions thiacloprid’s sorption on humic components will be most pronounced [79,80].

To determine the contribution of aromatic and aliphatic compounds in the neonicotinoids’ sorption, the dependence of log*K*_OC_ and the H/C atomic ratio for the analyzed soils was studied. The obtained results indicate that the H/C atomic ratios show a correlation with the affinity for neonicotinoid sorption, showing a negative trend between the log*K*_OC_ values and aliphaticity for the tested soils (*R*^2^ > 0.7932). The correlation between the log*K*_OC_ values and aliphaticity indicates that the greater the number of polar functional groups, the higher the sorption of acetamiprid, suggesting that aliphatic structures with polar functional groups which form the rubbery phase of the natural organic matter provide a suitable polar medium for acetamiprid binding. This is in accordance with the obtained order of nonlinearity for the sorption and desorption of neonicotinoids on the analyzed soils (soil S1 > soil S4 > soil S3 > soil S2). However, desorption from the rubbery phase is slow, pointing to the conclusion that the apparent irreversibility of sorption, i.e., the retention of acetamiprid on natural organic matter, is controlled by the aromatic structures that make up the glassy phase of the organic matter. Furthermore, it was determined that the ratios of the 1/n^sor^ value increase proportionally with the increase in H/C, indicating that with the increase in the aliphaticity of the soil organic phase, the sorption process becomes more of a distribution process.

Thus, the sorption/desorption of neonicotinoids does not solely depend on their distribution in the organic phase of the soil but is related to specific interactions between polar groups of neonicotinoid molecules and polar sites in the soil [81]. Although all three neonicotinoids possess polar parts of the molecule, their chemical behavior is quite different, which is manifested, for example, in drastically different solubility in water, with acetamiprid being the most soluble (4.25 g/L) and thiacloprid the least soluble (0.19 g/L) (Table 1). The distribution coefficient *K*_OW_, or log*K*_OW_, is important because it is directly proportional to the neonicotinoid’s tendency to be sorbed on the soil. In the case of thiacloprid with the highest value of log*K*_OW_ = 1.3 (Table 1), the affinity towards soil organic matter is extremely high, so this insecticide is practically completely sorbed on soil organic matter. So far, it has been demonstrated that soils whose organic matter is physically condensed and chemically reduced to a greater extent show a higher sorption capacity, and sorption isotherms are more nonlinear, with more pronounced sorption/desorption hysteresis and slower sorption [82]. Furthermore, although acetamiprid, imidacloprid, and thiacloprid belong to the same group of insecticides, they have different chemical structures. Namely, imidacloprid is a derivative of nitroguanidine and consists of a pyridine and imidazolidine ring, and it is characterized by a nitro group which is responsible for its insecticidal activity. Conversely, acetamiprid and thiacloprid are derivatives of *N*-cyanoamidine. Also, like imidacloprid, both *N*-cyanomidines have a pyridine ring but with a cyano group responsible for their insecticidal activity. However, acetamiprid and thiacloprid differ in chemical structure. In thiacloprid, the 1,3-thiazolidin-2-ylidene group is substituted with a (6-chloropyridin-3-yl) methyl group in the ring. In the case of acetamiprid, which is acetamidine, the amino hydrogens are substituted with (6-chloropyridin-3-yl) methyl and a methyl group, while the hydrogen attached to the imino nitrogen is replaced by a cyano group (Table 1). The main structural difference between acetamiprid and the other two neonicotinoids is that thiacloprid and imidacloprid possess thiazolidine and imidazolidine rings, that is, the thiazolidine and imidazolidine N of thiacloprid and imidacloprid carry a large aromatic unit, which could contribute to their low solubility in water [83].

The difference in the acid–base properties of neonicotinoids also contributes to their difference in soil sorption/desorption. Acetamiprid has a p*K*_a_ value of 0.7, the p*K*_a_ value of imidacloprid is 1.56 and 11.12, while thiacloprid does not dissociate (Table 1) [11,30]. The data indicate that acetamiprid is a strong acid, so at low pH values (acidic conditions), acetamiprid will be less dissociated and will remain in a neutral molecular form. In alkaline conditions, its dissociation begins, resulting in increased soil mobility. Imidacloprid undergoes very weak dissociation at soil pH values of 5–8 [53,84], while at very high pH values, dissociation increases, which may affect its bioavailability and interactions with soil constituents. Furthermore, neonicotinoids also contain nitrogen atoms in a similar environment: one *sp*-hybridized (nitrile group—N in acetamiprid and thiacloprid), two/three *sp*^2^-hybridized (one of which is a pyridine N in all neonicotinoids), and one *sp*^3^-hybridized N. The most pronounced difference can be observed for *sp*^3^ N, since it is located inside the ring structure and near S or N in thiacloprid and imidacloprid but not in acetamiprid. Therefore, it is likely that this *sp*^3^ N is responsible for protonation at a low pH. The protonation of *sp*^3^ N in an imidacloprid molecule will occur at very acidic pH values, usually below pH < 4 because the nitroguanidine moiety has relatively low basicity. The protonation of the *sp*^3^ N on the thiocarbamate moiety of thiacloprid will also occur under acidic conditions (pH < 5) because thiacloprid has a slightly higher basicity compared to the nitroguanidine moiety of imidacloprid but still requires an acidic environment for protonation.

In general, all neonicotinoids contain cyano or nitro groups that make up the negatively charged part of the molecule and are connected by aromatic structures that are deficient in π-electrons. Another partially negative part of the molecule is the nitrogen atom inside the pyridine ring, which has a lone electron pair capable of creating π-π or p-π electron donor–acceptor interactions (EDA) with aromatic parts of organic matter, which are rich in humic substances [71]. The oxygen in the –NO_2_ group in imidacloprid can bind with soil organic matter by forming H-bonds with functional groups of organic matter (carboxyl, hydroxyl, amide) [53,77]. Furthermore, N, S, and Cl heteroatoms in neonicotinoid molecules can act as hydrogen (H–) bond acceptors and form H-bonds with H-donating functional groups in the soil [85].

## 4. Materials and Methods

### 4.1. Soil Sampling and Physico-Chemical Soil Properties

**Figure 5:**
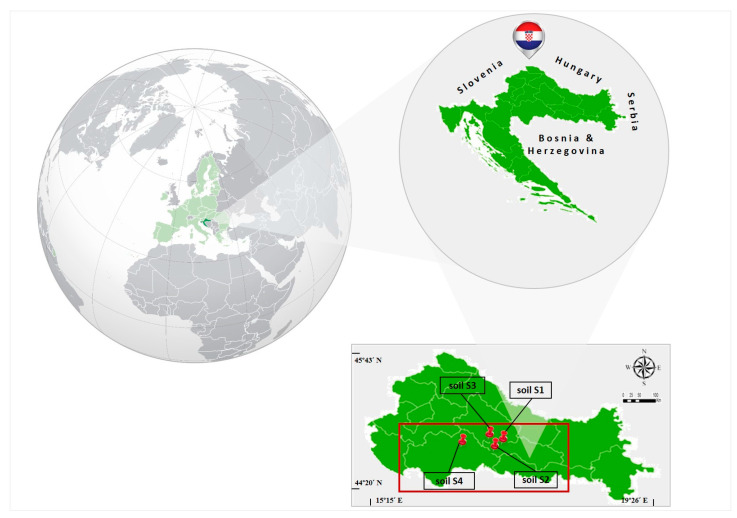
Location of the soil sampling sites on the Croatian geographical map. Counties (Požega-Slavonia and Sisak-Moslavina) where sampling was carried out are marked with a red box.

The field sites of this experiment were situated in two agricultural Croatian counties: Požega-Slavonia (the area around the cities of Lipik and Pakrac) and Sisak-Moslavina (the area around the city of Kutina). Topsoil samples were collected according to a Standard Sampling Procedure [86] on 0.5 ha (~50 × 100 m) from the four localities (soil S1—forest soil, city of Pakrac; soil S2—sediment soil from lake Raminac, city of Lipik; soil S3—agricultural soil, city of Pakrac; and soil S4—agricultural soil, city of Kutina) at depths of 0–30 cm using a stainless steel probe. Geographic Coordinate Systems (GCSs) of each sampling location are given in Table 2, while their positions on the geographic map are shown in Figure 5.

The soils were air-dried in the laboratory for one week at room temperature (20 ± 1 °C) with foreign material removed (small stones, leaves, twigs). Then, the soils were crushed in a porcelain mortar using a pestle and sieved through a 2 mm sieve. Soils from one locality prepared in this way were pooled, thoroughly mixed, and homogenized to form a composed sample and stored in plastic boxes prior to their use in sorption/desorption experiments. The soils were never treated with acetamiprid, imidacloprid, and thiacloprid, as verified by analyzing their residues in the soil.

The physico-chemical properties of experimental soils (Table 2) were determined according to standard laboratory procedure and were characterized as in Sinčić Modrić et al. [53].

### 4.2. Sorption/Desorption Equilibrium Experiments

Sorption/desorption experiments of acetamiprid, imidacloprid, and thiacloprid in experimental soil were carried out by the standard “batch” equilibrium method for testing the sorption and desorption processes of micropollutants, which is described in the OECD Technical Guideline 106 [87]. Stock standard solutions (1000 μg/mL) of each insecticide (analytical standards of acetamiprid, thiacloprid, and imidacloprid, purity > 99%, Dr. Ehrenstorfer GmbH, Augsburg, Germany) were prepared in acetonitrile (J.T. Baker, Deventer, Holland) at a concentration of 1000 μg/mL.

#### 4.2.1. Sorption Equilibrium Experiments of Acetamiprid, Thiacloprid, and Imidacloprid in Soil

For the sorption equilibrium experiments, 5 g (±0.01 g) of each soil and 25 mL of 0.01 M CaCl_2_ solutions (Kemika, Zagreb, Croatia) with acetamiprid, imidacloprid, and thiacloprid in the range from 0.1 to 25 mg/L were placed in 50 mL polypropylene centrifuge tubes. To maintain constant ionic strength of the insecticide solution as well as to facilitate the flocculation of the soil colloids, 0.01 M CaCl_2_ was used as a background electrolyte. The mixture of each insecticide solution and soil was agitated and equilibrated on a rotary shaker (Heidolph promax 2020, Schwabach, Germany) for 96 h (time interval determined by kinetic testing [53], at the temperature of the experiment (22 ± 2 °C)). After equilibration, suspensions were centrifuged at 4000 rpm for 3 min (Universal 320 R Hettich, Tuttlingen, Germany), and 1 mL of supernatant was removed, filtered (0.22 µm membrane filter; Merck, Darmstadt, Germany), and analyzed on HPLC-MS/MS.

For each series of experiments, to prevent losses of insecticide due to sorption on filters or centrifugal cuvettes, control samples were used: one control that did not contain insecticide solution but only soil and another control without soil but with the addition of insecticide.

The sorbed amount of insecticide in the soil at sorption equilibrium was calculated according to the Expression (1).

(1) $$q_{s}^{sor}(eq)=\frac{m_{s}^{sor}(eq)}{m_{soil}}=\frac{\left[\gamma_{0}-\gamma_{aq}^{sor}(eq)\right]{\cdot V}_{0}}{m_{soil}}$$

In Expression (1), $q_{s}^{sor}(eq)$ is the amount of sorbed insecticide in soil (mg/kg), $\gamma_{aq}^{sor}\left(eq\right)$ is the mass concentration of insecticide in solution (mg/L), $m_{s}^{sor}(eq)$ is the mass of insecticide sorbed in soil at sorption equilibrium (mg), and $V_{0}$ is the initial volume of the insecticide solution in contact with the soil (L).

#### 4.2.2. Desorption Equilibrium Experiments of Acetamiprid, Thiacloprid, and Imidacloprid in Soil

Equilibrium desorption experiments of selected insecticides were carried out on the same soil samples immediately after the sorption processes. The entire aqueous insecticide solution in equilibrium with the soil solid phase was removed and replaced with an equal volume (25 mL) of 0.01 M CaCl_2_ solution. Soils were resuspended using the vortex shaker, mixed, agitated, and equilibrated on a rotary shaker for 96 h at the temperature of the experiment. After equilibration, soil suspensions were centrifuged, and 1 mL of supernatant was filtered and analyzed for the presence of insecticides using HPLC-MS/MS.

The amount of remaining sorbed insecticide in the soil at desorption equilibrium was calculated according to Expression (2).

(2) $$q_{s}^{des}\left(eq\right)=\frac{m_{s}^{sor}\left(eq\right)-m_{aq}^{des}\left(eq\right)}{m_{soil}}$$

The total mass of insecticide desorbed from the soil at desorption equilibrium was determined through Expressions (3) and (4).

(3) $$m_{aq}^{des}\left(eq\right)=m_{m}^{des}\left(eq\right)\cdot \frac{V_{0}}{V_{r}^{F}}-m_{aq}^{A}$$

(4) $$m_{aq}^{A}\left(eq\right)=m_{aq}^{sor}\left(eq\right)\cdot \left(\frac{V_{0}-V_{R}}{V_{0}}\right)$$

In Expressions (2)–(4), $q_{s}^{des}\left(eq\right)$ is the amount of the remaining sorbed insecticide in the soil at desorption equilibrium (mg/kg), $m_{aq}^{A},$ $m_{aq}^{des}\left(eq\right)$, and $m_{m}^{des}\left(eq\right)$ are the mass of insecticide at sorption equilibrium left behind due to incomplete volume compensation (mg), the total mass of insecticide desorbed from the soil at desorption equilibrium (mg), and the mass of insecticide determined analytically in the aqueous phase at desorption equilibrium (mg), while $V_{r}^{F}$ and $V_{R}$ are the volume of solution taken for analysis at desorption equilibrium (mL) and the volume of the supernatant removed after reaching the sorption equilibrium and replaced with the same volume of 0.01 M CaCl_2_ (mL).

### 4.3. Analytical Methods

#### 4.3.1. Analysis of Ca^2+^, Mg^2+^, Na^+^, and K^+^ on AAS

Extracted cations Ca^2+^, Mg^2+^, Na^+^, and K^+^ were analyzed on AAS (Perkin Elmer Analyst, Waltham, MA, USA) according to the conditions listed in Table S4.

The linearity of the calibration curves, limits of detection (LODs), limits of quantification (LOQs), and other experimental conditions are described in more detail in our previous publication [53]. The amount of each ion was expressed in mg per kg of soil dry weight.

#### 4.3.2. Analysis of Insecticides on HPLC-MS/MS

A coupled system of liquid chromatography and mass spectrometry, HPLC-MS/MS (Exion LC, Concord, Ontario, Canada and 4500 QTRAP, AB Sciex, Framingham, MA, USA), was used for chromatographic separation and analysis of acetamiprid, imidacloprid, and thiacloprid. Insecticides were separated on a Phenomenex Kinetex C18 chromatographic column (Phenomenex, Torrance, CA, USA) with characteristics of 100 mm column length, 2.1 mm i.d., 2.6 µm, and 100 Å pore size. Chromatographic analysis of insecticide residues during sorption/desorption processes was carried out in accordance with the methods for determining pesticide residues in food according to ISO standards HRN EN ISO 12393-1:2013; 12393-2:2013; and 12393-3:2013 [88,89,90]. The methods were developed by the manufacturer of mass spectrometry instruments used in biomedicine and environmental protection “AB SCIEX” as methods for the determination of 203 pesticides (RUO-MKT-08-0918-A; Experimental Conditions for the Extraction and Analysis of 203 Pesticides from Food Samples; September, 2013). The mobile phase used consisted of the following solvents: 90% H_2_O, 10% CH_3_OH + 5 mM HCOONH_4_ (A), and 10% H_2_O, 90% CH_3_OH + 5 mM HCOONH_4_ (B), according to the gradient program shown in Table S5. The separation conditions were 0.4 mL/min, 30 µL injection volume, and a column temperature of 40 °C, and the total time of chromatographic analysis lasted 20 min. Under the mentioned chromatographic conditions, the retention times of imidacloprid, acetamiprid, thiacloprid were 4.74, 6.05, and 6.90 min.

The ionization source of the mass spectrometer was electrospray (ESI) operating in positive mode using multiple reaction monitoring (MRM) of the two most intense precursor–product ion transitions for each insecticide. More details on ESI ionization and MRM operating conditions are presented in Table S6. Analyst^®^ 1.6.1 software (AB Sciex, Framingham, MA, USA) support was used to process insecticide identification.

Calibration curves of each insecticide were performed with triplicate injection of standard solutions from 1 to 100 ng/mL (*R*^2^ > 0.9999 for all insecticides). LODs were 0.030, 0.028, and 0.024 ng/mL for acetamiprid, imidacloprid, and thiacloprid, respectively, while the LOQ was 0.1 ng/mL or below for all insecticides. Reproducibility was appropriate with a relative standard deviation (RSD) ≤5% in all cases, while recoveries were in the range from 94 to 106%.

### 4.4. Statistical Analysis

All data were presented as the mean of three determinations ± standard deviation. The software Wolfram Research Mathematica^®^ V.12.0 (Wolfram Research Co., Champaign, IL, USA) was used to estimate sorption/desorption parameters based on the experimental results using nonlinear regression models. In order to find out the most suitable isotherm models to represent the experimental data, three different error functions were used: the coefficient of multiple determination (*R*^2^), the standard error of the model (SRMSE, “Scaled Root Mean Squared Error”), and the chi-square test error (*χ*^2^ test error).

The software Statistica^®^ V. 14.0 (StatSoft, Inc., Tulsa, OK, USA) was used for statistical analysis of experimental results. Correlation matrix was applied to analyze the correlation between the soil physico-chemical characteristics and the sorption/desorption parameters obtained by mathematical modeling. Furthermore, in order to determine the dominant physico-chemical factors of the soil, a Principle Component Analysis (PCA) was performed, while their influence on the sorption/desorption parameters was examined by multiple regression. All statistical analyses were performed at a significance level of *p* < 0.05.

## 5. Conclusions

In line with our research results, and complementing existing studies, we propose that at lower concentrations (1 and 5 mg/L), acetamiprid molecules first occupy sorption sites in the rubbery aliphatic phase, while at higher concentrations (10–80 mg/L), they will fill sorption sites in the hydrophilic glassy regions. Imidacloprid will fill the sorption sites equally in the rubbery and glassy phases at lower and higher insecticide concentrations. Thiacloprid molecules will fill sorption sites in the glassy soil phase at low insecticide concentrations, while at higher concentrations, the rubbery soil phase will begin to be filled. The sorption area contains a limited number of high-energy sorption sites, and neonicotinoids first occupy sites at low concentrations, suggesting that at low concentrations, the sorption mechanism dominates the distribution. As indicated by the H/C, O/C, and (N + O)/C ratios, aromaticity will favor sorption, while a high concentration of hydrophilic carboxyl groups will suppress neonicotinoid sorption. The correlation between the log*K*_OC_ values and aliphaticity denotes that aliphatic structures with polar functional groups form a rubbery phase of organic matter that represents a suitable polar medium for acetamiprid binding. Furthermore, it was found that the ratios of 1/n^sor^ values increase proportionally with the increase in H/C, which indicates that with increasing aliphaticity of the soil organic phase, the sorption process becomes more of a distribution process. We assume that differences in the sorption/desorption behavior of neonicotinoids are manifested in *sp*^3^ N, since it is located inside the ring structure and close to S or N in thiacloprid and imidacloprid but not in acetamiprid. It can be inferred that this N atom is responsible for protonation at low pH values (pH < 5). Furthermore, thiacloprid has a thiazolidine nitrogen bearing a large aromatic unit, which we assume contributes to its low solubility in water. Through the N atom in the pyridine ring, neonicotinoids can form π-π or p-π electron donor–acceptor interactions (EDA) with aromatic parts of organic matter. Furthermore, N, S, and Cl heteroatoms in neonicotinoid molecules can act as hydrogen (H-) bond acceptors and form H-bonds with H-donating functional groups in the soil.

## Figures and Tables

**Table 1:** Chemical structure and physico-chemical properties of acetamiprid, imidacloprid, and thiacloprid [12].

Properties	Acetamiprid	Imidacloprid	Thiacloprid
Chemical structure	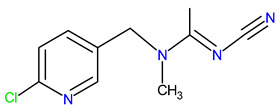	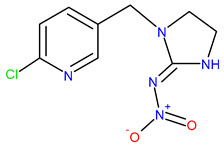	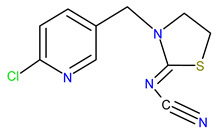
IUPAC name	N-[(6-chloropyridin-3-yl)methyl]-N′-cyano-N-methylethanimidamide	(NE)-N-[1-[(6-chloropyridin-3-yl)methyl]imidazolidin-2-ylidene]nitramide	[3-[(6-chloropyridin-3-yl)methyl]-1.3-thiazolidin-2-ylidene]cyanamide
Molecular formula	C_10_H_11_ClN_4_	C_9_H_10_ClN_5_O_2_	C_10_H_9_ClN_4_S
Molar mass (g/mol)	222.67	255.66	255.72
Melting point (°C)	98.9	144	136.0
Vapor pressure (mPa)	5.81 (25 °C)	4 × 10^−7^ (20 °C)	7.99 × 10^−7^ (20 °C)
Water solubility (g/L)	4.25 (25 °C)	0.61 (20 °C)	0.19 (20 °C)
*K*_OW_	6.31 (25 °C)	3.72 (21 °C)	18.20 (20 °C)
p*K*_a_	0.7	p*K*_a1_ = 1.56; p*K*_a2_ = 11.12	no dissociation
DT_50_ (day)	1–8.2	48–190	12–142
Hydrogen bond donor count	0	1	0
Hydrogen bond acceptor count	3	4	4
Topological polar surface area (Å^2^)	52.3	86.3	77.6

**Table 2:** Location, Geographic Coordinate System (GCS), and physical and chemical properties of soil samples collected from Požega-Slavonija (the area around the cities of Lipik and Pakrac) and Sisak-Moslavina county (the area around the city of Kutina).

Physico-Chemical Characteristics	Soil			
	S1	S2	S3	S4
Location	Pakrac	Lipik	Ploštine	Kutina
GCS	45°49′ N 17°08′ E	45°42′ N 17°13′ E	45°29′ N 17°07′ E	45°47′ N 16°48′ E
Textural classes	Clay loam	Clay loam	Clay loam	Clay loam
pH ^(a)^	4.94 (±0.11)	5.29 (±0.06)	5.25 (±0.04)	5.55 (±0.04)
HA ^(b)^ (cmol/kg)	13.39 (±1.02)	4.62 (±0.46)	4.59 (±0.44)	6.59 (±0.26)
CEC ^(c)^ (cmol/kg)	60.76 (±4.26)	48.28 (±1.54)	49.76 (±1.91)	49.59 (±1.69)
Clay (%)	30.75 (±1.25)	35.26 (±0.86)	36.62 (±0.67)	37.60 (±1.07)
Ca^2+^ (mg/100 g)	38.9 (±0.6)	25.7 (±1.9)	20.4 (±3.9)	23.0 (±2.9)
Mg^2+^ (mg/100 g)	450.8 (±33.8)	401.1 (±21.6)	447.0 (±34.8)	352.4 (±24.4)
Na^+^ (mg/100 g)	23.4 (±57.2)	30.9 (±4.5)	28.5 (±8.7)	31.5 (±5.4)
K^+^ (mg/100 g)	286.7 (±32.9)	315.1 (±46.4)	240.8 (±29.1)	449.5 (±5.4)
TOC ^(d)^ (%)	2.59 (±0.10)	1.06 (±0.15)	1.71 (±0.01)	2.21 (±0.05)
C_oxHa_^(e)^ (%)	0.56 (±0.06)	0.42 (±0.06)	0.74 (±0.14)	0.47 (±0.10)
C_oxFa_^(f)^ (%)	1.06 (±0.08)	0.32 (±0.03)	0.10 (±0.01)	0.70 (±0.03)
N (%)	0.221 (±0.009)	0.128 (±0.002)	0.175 (±0.002)	0.224 (±0.011)
C (%)	2.128 (±0.014)	0.946 (±0.018)	1.283 (±0.005)	1.728 (±0.040)
H (%)	0.595 (±0.005)	0.373 (±0.005)	0.456 (±0.009)	0.492 (±0.014)
S (%)	0.0242 (±0.0011)	0.0128 (±0.0004)	0.0174 (±0.0006)	0.0253 (±0.0008)
O (%)	97.032 (±0.09)	98.540 (±0.13)	98.068 (±0.06)	97.531 (±0.20)
Ratio H/C	3.33 (±0.02)	4.70 (±0.05)	4.24 (±0.07)	3.39 (±0.03)
Ratio N/C	0.089 (±0.004)	0.116 (±0.052)	0.117 (±0.013)	0.111 (±0.023)
Ratio S/C	0.0043 (±0.0003)	0.0051 (±0.0001)	0.0051 (±0.0006)	0.0055 (±0.0002)
Ratio O/C	34.23 (±0.02)	78.31 (±0.06)	57.38 (±0.03)	42.48 (±0.01)
Ratio (N + O)/C	34.32 (±0.06)	78.20 (±0.08)	57.38 (±0.04)	42.37 (±0.05)
Ratio E465/E665	8.20 (±0.31)	5.45 (±0.30)	6.76 (±0.09)	7.19 (±0.15)

^(a)^ Measured in soil + 0.01 M calcium chloride mixture (1:2.5. *w*/*V*); ^(b)^ hydrolytic acidity; ^(c)^ cation exchange capacity; ^(d)^ total organic carbon; ^(e)^ carbon of humic acids; ^(f)^ carbon of fulvic acids.

**Table 3:** Evaluated parameters with standard deviations and statistical indices for acetamiprid, imidacloprid, and thiacloprid sorption/desorption equilibrium processes in the tested soils (S1–S4) using the Freundlich nonlinear equilibrium model.

Sorption					Desorption			
Fitted/Statistical Parameter	S1	S2	S3	S4	S1	S2	S3	S4
Acetamiprid								
*K*_F_^sor/des (a,b)^ [(mg/kg)/(mg/L)]^1/n^	11.31 (±1.51)	3.56 (±0.28)	4.98 (±0.67)	6.46 (±1.09)	15.27 (±2.29)	6.73 (±0.90)	9.49 (±0.81)	11.65 (±1.31)
1/n^sor/des (c,d)^	0.848 (±0.030)	0.772 (±0.011)	0.777 (±0.026)	0.784 (±0.031)	0.848 (±0.039)	0.765 (±0.035)	0.732 (±0.025)	0.779 (±0.030)
*R* ^2 (e)^	0.9991	0.9999	0.9994	0.9990	0.9987	0.9988	0.9985	0.9992
SRMSE ^(f)^	0.0422	0.0165	0.0360	0.0449	0.0641	0.0542	0.0395	0.0491
err-% ^(g)^	3.36	1.31	2.87	3.57	5.09	4.31	3.15	3.90
m ^(h)^	4 (*χ*^2^_tab_ = 9.488 at *p* = 0.05)							
Imidacloprid								
*K*_F_^sor/des (a,b)^ [(mg/kg)/(mg/L)]^1/n^	18.61 (±3.01)	5.68 (±0.94)	6.83 (±1.67)	10.53 (±0.96)	26.70 (±2.67)	10.76 (±2.33)	13.50 (±1.12)	20.93 (±2.48)
1/n^sor/des (c,d)^	0.895 (±0.033)	0.740 (±0.019)	0.757 (±0.043)	0.704 (±0.020)	0.881 (±0.028)	0.738 (±0.059)	0.749 (±0.027)	0.699 (±0.037)
*R* ^2 (e)^	0.9985	0.9994	0.9979	0.9995	0.9977	0.9979	0.9953	0.9991
SRMSE ^(f)^	0.0560	0.0328	0.0649	0.0314	0.0486	0.0926	0.0414	0.0667
err-% ^(g)^	3.48	2.61	5.16	2.50	3.87	7.36	3.29	5.31
m ^(h)^	4 (*χ*^2^_tab_ = 9.488 at *p* = 0.05)							
Thiacloprid								
*K*_F_^sor/des (a,b)^ [(mg/kg)/(mg/L)]^1/n^	32.60 (±4.42)	6.71 (±3.13)	8.54 (±2.44)	13.74 (±1.49)	64.45 (±4.81)	17.26 (±2.74)	29.60 (±4.86)	46.51 (±5.21)
1/n^sor/des (c,d)^	0.755 (±0.039)	0.829 (±0.049)	0.791 (±0.042)	0.665 (±0.024)	0.753 (±0.039)	0.826 (±0.049)	0.706 (±0.058)	0.640 (±0.053)
*R* ^2 (e)^	0.9999	0.9959	0.9978	0.9999	0.9959	0.9978	0.9969	0.9949
SRMSE ^(f)^	0.0657	0.0944	0.0736	0.0408	0.0652	0.0753	0.0821	0.0993
err-% ^(g)^	5.22	7.51	5.85	3.24	5.18	5.99	6.55	7.90
m ^(h)^	4 (*χ*^2^_tab_ = 9.488 at *p* = 0.05)							

^(a,b), (c,d)^ Sorption/desorption parameters obtained by modelling with Freundlich model; ^(e)^ coefficient of multiple determination; ^(f)^ Scaled Root Mean Squared Error; ^(g)^ minimum error level of *χ*^2^ test; ^(h)^ degrees of freedom = number of measurements − number of model parameters.

**Table 4:** Values of organic carbon partition coefficient (*K*_OC_), Gibbs free energy (ΔG), and hysteresis coefficients (*H* and *λ*) with standard deviations for acetamiprid, imidacloprid, and thiacloprid sorption/desorption equilibrium processes in the tested soils (S1–S4).

Parameters	Acetamiprid				Imidacloprid				Thiacloprid			
	S1	S2	S3	S4	S1	S2	S3	S4	S1	S2	S3	S4
*K*_OC_ (L/kg)	436.85 (±47.86)	284.79 (±21.86)	292.16 (±48.87)	292.46 (±21.84)	718.51 (±24.11)	454.52 (±42.94)	399.52 (±6.78)	476.90 (±44.51)	1258.10 (±24.00)	537.09 (±10.87)	499.49 (±83.05)	621.38 (±19.55)
ΔG (kJ/mol)	−14.81 (±0.27)	−13.77 (±0.22)	−13.82 (±4.09)	−13.83 (±1.82)	−16.03 (±0.81)	−14.91 (±0.23)	−14.56 (±0.41)	−15.03 (±2.27)	−17.39 (±0.46)	−15.32 (±0.49)	−15.13 (±4.07)	−15.68 (±0.77)
*H*	0.904 (±0.032)	0.991 (±0.004)	0.940 (±0.011)	0.913 (±0.001)	0.967 (±0.001)	0.869 (±0.015)	0.862 (±0.003)	0.869 (±0.012)	0.988 (±0.013)	0.996 (±0.006)	0.891 (±0.033)	0.905 (±0.020)
*λ*	0.046 (±0.016)	0.004 (±0.002)	0.027 (±0.006)	0.040 (±0.001)	0.016 (±0.001)	0.059 (±0.009)	0.063 (±0.002)	0.057 (±0.007)	0.005 (±0.001)	0.002 (±0.001)	0.051 (±0.018)	0.040 (±0.009)

**Table 5:** Matrix correlations analysis for soil properties and parameters obtained by the Freundlich model for acetamiprid sorption and desorption in the tested soils (S1–S4). Bold typeface indicates statistically significant correlations at *p* < 0.05.

Variable	$K_{F}^{sor\;(f)}$	1/n^sor (g)^	$K_{F}^{des\;(h)}$	1/n^des (i)^	*K*_OC_ ^(j)^	ΔG ^(k)^	*H* ^(l)^	*λ* ^(m)^
pH	−0.63	−0.69	−0.46	−0.63	**−0.77** (*p* = 0.025)	**0.75** (*p* = 0.031)	0.17	−0.21
HA ^(a)^	**0.96** (*p* < 0.001)	**0.88** (*p* = 0.004)	**0.92** (*p* = 0.001)	0.35	**0.90** (*p* = 0.002	**−0.89** (*p* = 0.003)	−0.65	0.68
CEC ^(b)^	**0.94** (*p* < 0.001)	**0.89** (*p* = 0.003)	**0.87** (*p* = 0.005)	0.40	**0.92** (*p* = 0.001)	**−0.91** (*p* = 0.002)	−0.60	0.64
Clay	**−0.76** (*p* = 0.028)	**−0.79** (*p* = 0.019)	−0.62	−0.66	**−0.86** (*p* = 0.006)	**0.85** (*p* = 0.008)	0.25	−0.30
TOC ^(c)^	**0.91** (*p* = 0.002)	**0.74** (*p* = 0.035)	**0.98** (*p* < 0.001)	−0.08	**0.71** (*p* = 0.049)	−0.71	**−0.90** (*p* = 0.002)	**0.91** (*p* = 0.002)
C_oxHa_^(d)^	0.10	0.08	0.09	−0.20	0.08	−0.07	−0.28	0.27
C_oxFa_^(e)^	**0.87** (*p* = 0.005)	**0.76** (*p* = 0.030)	**0.89** (*p* = 0.003)	0.21	**0.75** (*p* = 0.031)	**−0.75** (*p* = 0.032)	−0.64	0.67
Ratio E465/E665	**0.91** (*p* = 0.001)	**0.75** (*p* = 0.030)	**0.97** (*p* < 0.001)	−0.07	**0.73** (*p* = 0.040)	**−0.72** (*p* = 0.042)	**−0.91** (*p* = 0.002)	**0.91** (*p* = 0.002)
Ratio H/C	**−0.82** (*p* = 0.013)	−0.63	**−0.93** (*p* = 0.001)	0.19	−0.58	0.58	**0.88** (*p* = 0.003)	**−0.88** (*p* = 0.003)
Ratio N/C	**−0.95** (*p* < 0.001)	**−0.86** (*p* = 0.006)	**−0.92** (*p* = 0.001)	−0.33	**−0.87** (*p* = 0.005)	**0.86** (*p* = 0.006)	0.64	−0.67
Ratio S/C	**−0.78** (*p =* 0.023)	**−0.80** (*p =* 0.018)	−0.64	−0.56	**−0.86** (*p* = 0.06)	**0.84** *(p* = 0.09)	0.36	−0.39
Ratio O/C	**−0.86** (*p* = 0.006)	−0.68	**−0.95** (*p* < 0.001)	0.19	−0.63	0.63	**0.93** (*p* = 0.001)	**−0.93** (*p* = 0.001)
Ratio (N + O)/C	**−0.86** (*p* = 0.006)	−0.68	**−0.95** (*p* < 0.001)	0.19	−0.63	0.63	**0.93** (*p* = 0.001)	**−0.93** (*p* = 0.001)

^(a)^ Hydrolytic acidity; ^(b)^ cation exchange capacity; ^(c)^ total organic carbon; ^(d)^ carbon of humic acids; ^(e)^ carbon of fulvic acids; ^(f), (g), (h), (i)^ parameters obtained by modelling with Freundlich model; ^(j)^ organic carbon partition coefficient; ^(k)^ molar free Gibbs energy; ^(l), (m)^ hysteresis coefficients.

**Table 6:** Matrix correlations analysis for soil properties and parameters obtained by the Freundlich model for imidacloprid sorption and desorption in the tested soils (S1–S4). Bold typeface indicates statistically significant correlations at *p* < 0.05.

Variable	$K_{F}^{sor\;(f)}$	1/n^sor (g)^	$K_{F}^{des\;(h)}$	1/n^des (i)^	*K*_OC_ ^(j)^	ΔG ^(k)^	*H* ^(l)^	*λ* ^(m)^
pH	−0.61	**−0.94** (*p* = 0.001)	−0.27	**−0.91** (*p* = 0.001)	**−0.71** (*p* = 0.047)	0.67	**−0.82** (*p* = 0.013)	**0.78** (*p* = 0.022)
HA ^(a)^	**0.98** (*p* < 0.001)	**0.87** (*p* = 0.005)	**0.85** (*p* = 0.007)	**0.93** (*p* = 0.001)	**0.97** (*p* < 0.001)	**−0.96** (*p* < 0.001)	**0.97** (*p* < 0.001)	**−0.97** (*p* < 0.001)
CEC ^(b)^	**0.95** (*p* < 0.001)	**0.94** (*p* = 0.001)	**0.77** (*p* = 0.027)	**0.98** (*p* < 0.001)	**0.94** (*p* = 0.001)	**−0.92** (*p* = 0.001)	**0.98** (*p* < 0.001)	**−0.96** (*p* < 0.001)
Clay	**−0.78** (*p* = 0.023)	**−0.95** (*p* < 0.001)	−0.48	**−0.97** (*p* < 0.001)	**−0.89** (*p* = 0.003)	**0.87** (*p* = 0.005)	**−0.94** (*p* = 0.001)	**0.92** (*p* = 0.001)
TOC ^(c)^	**0.92** (*p* = 0.001)	0.58	**0.99** (*p* < 0.001)	0.66	**0.75** (*p* = 0.030)	**−0.75** (*p* = 0.033)	**0.73** (*p* = 0.039)	**−0.75** (*p* = 0.034)
C_oxHa_^(d)^	0.01	0.22	−0.02	0.14	−0.15	0.20	−0.01	0.04
C_oxFa_^(e)^	**0.93** (*p* = 0.001)	0.62	**0.91** (*p* = 0.002)	**0.71** (*p* = 0.047)	**0.90** (*p* = 0.003)	**−0.91** (*p* = 0.002)	**0.83** (*p* = 0.011)	**−0.85** (*p* = 0.008)
Ratio E465/E665	**0.90** (*p* = 0.002)	0.65	**0.94** (*p* = 0.001)	0.70	**0.73** (*p* = 0.042)	**−0.71** (*p* = 0.050)	**0.74** (*p* = 0.037)	**−0.74** (*p* = 0.035)
Ratio H/C	**−0.84** (*p* = 0.009)	−0.39	**−0.98** (*p* < 0.001)	−0.49	−0.66	0.67	−0.60	0.63
Ratio N/C	**−0.98** (*p* < 0.001)	**−0.81** (*p* = 0.016)	**−0.87** (*p* = 0.004)	**−0.87** (*p* < 0.001)	**−0.97** (*p* < 0.001))	**0.96** (*p* < 0.001))	**−0.94** (*p* < 0.001)	**0.95** (*p* < 0.001)
Ratio S/C	**−0.76** (*p* = 0.027)	**−0.98** (*p* < 0.001)	−0.47	**−0.97** (*p* < 0.001)	**−0.82** (*p* = 0.013)	**0.78** (*p* = 0.022)	**−0.90** (*p* = 0.002)	**0.88** (*p* = 0.004)
Ratio O/C	**−0.86** (*p* = 0.006)	−0.50	**−0.97** (*p* < 0.001)	−0.57	−0.66	0.65	−0.64	0.65
Ratio (N + O)/C	**−0.86** (*p* = 0.007)	−0.50	**−0.96** (*p* < 0.001)	−0.57	−0.66	0.65	−0.64	0.65

^(a)^ Hydrolytic acidity; ^(b)^ cation exchange capacity; ^(c)^ total organic carbon; ^(d)^ carbon of humic acids; ^(e)^ carbon of fulvic acids; ^(f), (g), (h), (i)^ parameters obtained by modelling with Freundlich model; ^(j)^ organic carbon partition coefficient; ^(k)^ molar free Gibbs energy; ^(l), (m)^ hysteresis coefficients.

**Table 7:** Matrix correlations analysis for soil properties and parameters obtained by the Freundlich model for thiacloprid sorption and desorption in the tested soils (S1–S4). Bold typeface indicates statistically significant correlations at *p* < 0.05.

Variable	$K_{F}^{sor\;(f)}$	1/n^sor (g)^	$K_{F}^{des\;(h)}$	1/n^des (i)^	*K*_OC_ ^(j)^	ΔG ^(k)^	*H* ^(l)^	*λ* ^(m)^
pH	−0.69	−0.42	−0.41	−0.56	**−0.76** (*p* = 0.029)	0.70	−0.54	0.50
HA ^(a)^	**0.99** (*p* < 0.001)	−0.25	**0.90** (*p* = 0.002)	−0.01	**0.99** (*p* < 0.001)	**−0.98** (*p* < 0.001)	0.42	−0.44
CEC ^(b)^	**0.98** (*p* < 0.001)	−0.11	**0.85** (*p* = 0.008)	0.09	**0.98** (*p* < 0.001)	**−0.96** (*p* < 0.001)	0.41	−0.41
Clay	**−0.84** (*p* = 0.009)	−0.22	−0.58	−0.48	**−0.91** (*p* = 0.002)	**0.87** (*p* = 0.005)	−0.70	0.68
TOC ^(c)^	**0.88** (*p* = 0.004)	−0.63	**0.99** (*p* < 0.001)	−0.51	**0.80** (*p* = 0.018)	**−0.81** (*p* = 0.015)	−0.05	0.01
C_oxHa_^(d)^	0.03	0.15	0.07	−0.17	−0.03	0.09	−0.55	0.59
C_oxFa_^(e)^	**0.90** (*p* = 0.002)	−0.49	**0.89** (*p =* 0.003)	−0.19	**0.88** (*p* = 0.004)	**−0.91** (*p* = 0.002)	0.40	−0.45
Ratio E465/E665	**0.87** (*p* = 0.005)	−0.54	**0.97** (*p* < 0.001)	−0.47	**0.79** (*p* = 0.020)	**−0.79** (*p* = 0.020)	−0.11	0.08
Ratio H/C	**−0.79** (*p* = 0.022)	**0.77** (*p* = 0.026)	**−0.95** (*p* < 0.001)	0.63	−0.69	**0.72** (*p* = 0.042)	0.10	−0.04
Ratio N/C	**−0.98** (*p* < 0.001)	0.32	**−0.91** (*p* = 0.002)	0.04	**−0.97** (*p* < 0.001)	**0.98** (*p* < 0.001)	−0.44	0.47
Ratio S/C	**−0.83** (*p* = 0.01)	−0.23	−0.60	−0.38	**−0.87** (*p* = 0.005)	**0.82** (*p* = 0.012)	−0.49	0.46
Ratio O/C	**−0.81** (*p* = 0.015)	0.68	**−0.97** (*p* < 0.001)	0.62	**−0.71** (*p* = 0.05)	**0.72** (*p* = 0.04)	0.19	−0.14
Ratio (N + O)/C	**−0.81** (*p* = 0.015)	0.68	**−0.97** (*p* < 0.001)	0.62	**−0.71** (*p* = 0.05)	**0.72** (*p* = 0.04)	0.19	−0.14

^(a)^ Hydrolytic acidity; ^(b)^ cation exchange capacity; ^(c)^ total organic carbon; ^(d)^ carbon of humic acids; ^(e)^ carbon of fulvic acids; ^(f), (g), (h), (i)^ parameters obtained by modelling with Freundlich model; ^(j)^ organic carbon partition coefficient; ^(k)^ molar free Gibbs energy; ^(l), (m)^ hysteresis coefficients.

**Table 8:** Results of principal component analysis (PCA) of physical and chemical properties of tested soils and evaluated parameters for acetamiprid, imidacloprid, and thiacloprid sorption/desorption equilibrium processes in the tested soils (S1–S4) obtained by the Freundlich nonlinear equilibrium model. The contribution of the variables was represented in the four principal components (PC1–PC4) by eigenvectors.

Principal Component	PC1	PC2	PC3	PC4
Eigenvalue	11.52	3.94	2.58	1.70
% Total variance	54.87	18.75	12.28	8.08
Cumulative %	54.87	73.62	85.90	93.98
Eigenvectors				
HA ^(a)^	0.289	−0.031	0.071	0.073
pH	−0.203	0.287	−0.072	0.305
CEC ^(b)^	0.340	−0.078	0.095	−0.066
clay	−0.243	0.251	−0.026	0.057
TOC ^(c)^	0.258	0.207	0.128	0.082
C_oxHa_^(d)^	0.019	0.058	0.206	−0.647
C_oxFa_^(e)^	0.259	0.064	0.015	0.333
Ratio E465/E665	0.258	0.184	0.161	−0.059
Ratio H/C	−0.227	−0.290	−0.096	−0.221
Ratio N/C	−0.284	0.009	−0.049	−0.166
Ratio S/C	−0.242	0.212	−0.092	0.248
Ratio O/C	−0.238	−0.255	−0.146	−0.043
Ratio (N + O)/C	−0.238	−0.254	−0.146	−0.043
*K*_F_^sor (f)^	0.287	0.059	−0.272	−0.087
1/n^sor (g)^	0.112	−0.360	0.304	−0.010
*K*_F_^des (h)^	0.188	0.162	−0.406	−0.133
1/n^des (i)^	0.127	−0.360	0.275	0.154
*K*_OC_ ^(j)^	0.220	0.012	−0.381	−0.137
ΔG ^(k)^	−0.194	0.002	0.366	0.223
*λ* ^(l)^	−0.084	0.311	0.284	−0.217
*H* ^(m)^	0.089	−0.323	−0.255	0.220

^(a)^ Hydrolytic acidity; ^(b)^ cation exchange capacity; ^(c)^ total organic carbon; ^(d)^ carbon of humic acids; ^(e)^ carbon of fulvic acids; ^(f), (g), (h), (i)^ parameters obtained by modelling with Freundlich model; ^(j)^ organic carbon partition coefficient; ^(k)^ molar free Gibbs energy; ^(l), (m)^ hysteresis coefficients.

**Table 9:** Prediction of sorption/desorption parameters $K_{F}^{sor},\;\;K_{F}^{des},\;\;1/n^{sor},\;and\;1/n^{des}$ from physico-chemical soil properties: cation exchange capacity (CEC), humic acid content (C_oxHa_), and ratio H/C represented by multiple linear regression.

Insecticide	Regression Equation	*R* ^2^	*p*
Acetamiprid	$K_{F}^{sor}=0.417\times CEC-1.859\times ratioH/C+0.467\times C_{oxHa}-8.160$	0.937	0.0024
	$1/n^{sor}=0.006\times CEC-0.005\times ratioH/C-0.008\times C_{oxHa}+0.520$	0.642	0.0736
	$K_{F}^{des}=0.358\times CEC-4.497\times ratioH/C+1.963\times C_{oxHa}+9.489$	0.985	0.0001
	$1/n^{des}=0.005\times CEC+0.040\times ratioH/C-0.082\times C_{oxHa}+0.346$	0.412	0.1863
Imidacloprid	$K_{F}^{sor}=0.727\times CEC-3.247\times ratioH/C-3.420\times C_{oxHa}-12.911$	0.986	0.0001
	$1/n^{sor}=0.017\times CEC+0.047\times ratioH/C+0.024\times C_{oxHa}-0.319$	0.943	0.0020
	$K_{F}^{des}=0.256\times CEC-8.740\times ratioH/C-0.919\times C_{oxHa}+41.082$	0.991	<0.0001
	$1/n^{des}=0.023\times CEC+0.048\times ratioH/C-0.025\times C_{oxHa}-0.692$	0.993	<0.0001
Thiacloprid	$K_{F}^{sor}=1.713\times CEC-4.087\times ratioH/C-6.811\times C_{oxHa}-54.201$	0.998	<0.0001
	$1/n^{sor}=0.009\times CEC+0.139\times ratioH/C+0.003\times C_{oxHa}-0.253$	0.752	0.0356
	$K_{F}^{des}=1.342\times CEC-22.126\times ratioH/C+7.501\times C_{oxHa}+51.968$	0.999	<0.0001
	$1/n^{des}=0.017\times CEC+0.185\times ratioH/C-0.244\times C_{oxHa}-0.711$	0.962	0.0009

## Data Availability

Data is contained within the article and Supplementary Materials.

## Supplementary Materials

The following supporting information can be downloaded at https://www.mdpi.com/article/10.3390/ijms25115700/s1. References [66,91,92] are mentioned in Supplementary Materials.
