# Supplementary material for: Those That Remain Caught in the “Organic Matter Trap”: Sorption/Desorption Study for Levelling the Fate of Selected Neonicotinoids
Source: Int J Mol Sci. 2024 May 23;25(11):5700. doi: 10.3390/ijms25115700 (PMC11172031; doi:10.3390/ijms25115700)
Supplement: Supplementary file 1 [file ijms-25-05700-s001.zip › ijms-3024603-supplementary.pdf]

Supplementary Materials

Those That Remain Caught in the "Organic Matter Trap": Sorption/Desorption Study for Levelling the Fate of Selected Neonicotinoids

Gordana Sinčić Modrić [1,†], Jelena Marinić [2,†], Romano Karleuša [2], Igor Dubrović [1], Przemysław Kosobucki [3] and Dalibor Broznić [2,*]

[1] Department of Environmental Health, Teaching Institute of Public Health of Primorje-Gorski Kotar County, Krešimirova 52a, 51000 Rijeka, Croatia; gordana.modric@zzjzpgz.hr (G.S.M.); igor.dubrovic@zzjzpgz.hr (I.D.)
[2] Department for Medical Chemistry, Biochemistry and Clinical Chemistry, Faculty of Medicine, University of Rijeka, Brace Branchetta 20, 51000 Rijeka, Croatia; jelena.marinic@uniri.hr (J.M.); romano.karleusa@uniri.hr (R.K.)
[3] Department of Food Analysis and Environmental Protection, Faculty of Chemical Technology and Engineering, University of Science and Technology of Bydgoszcz, 3 Seminaryjna Street, 85-326 Bydgoszcz, Poland; p.kosobucki@pbs.edu.pl
* Correspondence: dalibor.broznic@uniri.hr
† The authors contributed equally to this work.

4.5. Data analysis

The sorption and desorption equilibrium processes of acetamiprid, imidacloprid and thiacloprid in analyzed soils were represented by sorption isotherms, *i.e.* mathematical models of a theoretical or empirical nature describing the dependency of the sorbed insecticide amount per unit mass of the soil, $q^{\mathrm{sor/des}}$ (mg/kg) and the equilibrium insecticide concentration in the aqueous phase, $\gamma_{\mathrm{eq}}^{\mathrm{sor/des}}$(mg/L) at constant temperature. Three sorption isotherms, Freundlich's, Langmuir's and Temkin's isotherms were used for modelling of insecticide transport and containment in the soil matrix.

4.5.1. Freundlich's isotherm

The Freudlich isotherm describes non-ideal reversible chemisorption and is not limited to monolayer formation as is the case with the Langmuir isotherm. Freundlich isotherms are observed when sorption takes place on heterogeneous surfaces, they are non-linear, sorption decreases with increasing concentration. The coefficients (K_F and 1/n) in the Freundlich sorption/desorption isotherm can be determined by direct non-linear regression of experimental data according to the expression (S1).

$$q^{\mathrm{sor/des}} = K_{\mathrm{F}}^{\mathrm{sor/des}} \gamma_{\mathrm{eq}}^{1/n} \quad \text{(S1)}$$

In the expression (S1) $q^{\mathrm{sor/des}}$ is the insecticide amount retained by the soil (mg/kg), γ_{eq} is equilibrium concentration of insecticide in the solution (mg/L), while $K_{\mathrm{F}}^{\mathrm{sor/des}}$ and $1/n$ are Freundlich constants referring to the relative sorption/desorption capacity or intensity.

4.5.2. Langmuir's isotherm

The Langmuir isotherm describes insecticide sorption on a homogeneous soil surface with the maximum sorbed amount of molecules in a monolayer with a thickness equal to the dimension of one molecule without lateral interactions. In this case, the sorption rate is proportional to the uncovered surface of the sorbent, while the desorption rate depends to the surface that is already covered by a monomolecular layer. Therefore, the sorption energy is proportional to the soil surface and independent of the degree of surface coverage. In addition, sorption is localized at the sites of remaining sorbed insecticide molecules until their desorption. Desorption occurs when the sorbed molecules gain enough energy to return to the gaseous state or solution and then desorb. The Langmuir isotherm is defined by expression (S2).

$$q^{\mathrm{sor/des}} = \frac{q_{\mathrm{max}}^{\mathrm{sor/des}} K_{\mathrm{L}}^{\mathrm{sor/des}} \gamma_{\mathrm{eq}}}{1 + K_{\mathrm{L}}^{\mathrm{sor/des}} \gamma_{\mathrm{eq}}} \quad \text{(S2)}$$

In expression (S2) $q_{\mathrm{max}}^{\mathrm{sor/des}}$ is maximum soil sorption/desorption capacity for monolayer formation (mg/kg), $K_{\mathrm{L}}^{\mathrm{sor/des}}$is Langmuir constant (L/mg) depended on the bond strength between soil surface and insecticide molecule .

4.5.3. Temkin's isotherm

Temkin model assumes that the heat of sorption/desorption of all molecules in the layer decreases linearly with coverage rather than logarithmically. Model contains a factor that explicitly takes into account insecticide-soil interactions, ignoring extremely low and high concentration values. Thus, the Temkin's isotherm is valid only for the middle range of insecticide concentrations [1]. The Temkin isotherm shows the amount of the sorbed/desorbed insecticide quantity ($q^{\mathrm{sor/des}}$) in relation to ($\ln\gamma_{\mathrm{eq}}$)(expression S3).

$$q^{\mathrm{sor/des}} = \frac{\mathrm{RT}}{\mathrm{b}} \ln\left(K_{\mathrm{T}}^{\mathrm{sor/des}} \gamma_{\mathrm{eq}}\right) \quad \text{(S3)}$$

The linearized form of expression (S3) is given by expression (S4).

$$q^{\mathrm{sor/des}} = B_1 \ln K_{\mathrm{T}}^{\mathrm{sor/des}} + B_1 \ln\gamma_{\mathrm{eq}} \quad \text{(S4)}$$

In the expressions (7 and 8) $B_1$and b are constants that determines the characteristics of sorption/desorption energy (J/mol) and $K_{\mathrm{T}}^{\mathrm{sor/des}}$ is a bond constant in the equilibrium state (responsible for maximum bond energy) (L/mg).

Organic carbon partition coefficient, K_{OC} (L/kg) normalized to the soil organic matter was calculated according to the expression (S5).

$$K_{\mathrm{OC}} = 100 \times \frac{K_{\mathrm{F}}^{\mathrm{sor}}}{f_{\mathrm{OC}}} \quad \text{(S5)}$$

In expression (S5) f_{OC} represent the amount of the OC content per unit mass of the soil (%). Effect of K_{OC} on the molar free Gibbs energy, ΔG (kJ/mol) is represented by expression (S6).

$$\Delta\mathrm{G} = -RT \ln K_{\mathrm{OC}} \quad \text{(S6)}$$

In expression (S6), T is the absolute temperature (K), and R is the universal gas constant (8.314 J/molK).

The discrepancy between sorption and desorption isotherms, *i.e.* the comparison of hysteresis extent of analyzed insecticides in different soils is shown by hysteresis indices, H and λ. According to the methods proposed by Cox et al. [2] and Selim and Zhu [3] indices were calculated using expressions (S7) and (S8).

$$H = 100 \times \frac{1/n^{\mathrm{des}}}{1/n^{\mathrm{sor}}} \quad \text{(S7)}$$

$$\lambda = 100 \times \left(\frac{1/n^{\mathrm{sor}} + 1}{1/n^{\mathrm{des}} + 1} - 1\right) \quad \text{(S8)}$$

In expressions (S7) and (S8) $1/n^{\mathrm{sor}}$ and $1/n^{\mathrm{des}}$ are previously defined Freundlich sorption/desorption coefficients of nonlinearity.

Table S1. Evaluated parameters with standard deviations and statistical indices for acetamiprid, imidacloprid and thiacloprid sorption/desorption equlibrium processes in the tested soils (S1 – S4) using Langmuir nonlinear equilibrium model.

	Sorption				Desorption			
Fitted/statistical parameter	S1	S2	S3	S4	S1	S2	S3	S4
				Acetamiprid				
$K_L^{sor/des}$ (a, b) (L/mg)	0.031 (±0.005)	0.053 (±0.006)	0.062 (±0.011)	0.075 (±0.027)	0.329 (±0.329)	0.652 (±0.034)	0.848 (±0.822)	0.432 (±0.381)
$q_m^{sor/des}$ (c, d) (mg/kg)	408.33 (±11.78)	70.44 (±1.40)	88.89 (±0.56)	97.24 (±14.55)	110.70 (±75.28)	21.23 (±0.06)	25.46 (±0.92)	59.06 (±8.30)
R^2 (e)	0.9169	0.9329	0.9039	0.9450	0.8883	0.8584	0.9206	0.8920
SRMSE (f)	0.3070	0.2370	0.3042	0.2266	0.3486	0.4737	0.2764	0.3360
err-% (g)	24.41	18.84	24.19	18.02	27.72	37.67	21.98	26.72
m (h)				4 (χ^2_{tab} = 9.488 at p = 0.05)				
				Imidacloprid				
$K_L^{sor/des}$ (a, b) (L/mg)	0.099 (±0.013)	0.048 (±0.006)	0.034 (±0.009)	0.107 (±0.013)	0.200 (±0.025)	1.075 (±1.051)	1.244 (±1.252)	1.985 (±2.107)
$q_m^{sor/des}$ (c, d) (mg/kg)	243.83 (±37.39)	122.24 (±8.43)	212.72 (±52.72)	148.84 (±29.19)	168.08 (±1.99)	26.01 (±0.43)	29.78 (±1.00)	36.92 (±2.60)
R^2 (e)	0.8957	0.9163	0.9176	0.9328	0.8877	0.8723	0.8620	0.8963
SRMSE (f)	0.3506	0.2856	0.2810	0.2425	0.3593	0.4173	0.4399	0.3332
err-% (g)	27.88	22.71	22.35	19.28	28.57	33.19	34.98	26.49
m (h)				4 (χ^2_{tab} = 9.488 at p = 0.05)				
				Thiacloprid				
$K_L^{sor/des}$ (a, b) (L/mg)	0.478 (±0.139)	0.022 (±0.010)	0.130 (±0.005)	0.135 (±0.062)	2.875 (±0.176)	0.200 (±0.016)	2.278 (±0.458)	6.113 (±3.266)
$q_m^{sor/des}$ (c, d) (mg/kg)	125.00 (±19.64)	118.52 (±60.48)	74.39 (±30.77)	147.73 (±48.21)	74.92 (±1.19)	53.62 (±0.20)	45.09 (±2.01)	48.60 (±4.16)
R^2 (e)	0.9452	0.9340	0.9134	0.8978	0.8524	0.8999	0.8563	0.8600
SRMSE (f)	0.2334	0.2556	0.2986	0.3289	0.4506	0.3269	0.4477	0.4429
err-% (g)	18.56	20.32	23.74	26.15	35.83	26.00	35.60	35.22
m (h)				4 (χ^2_{tab} = 9.488 at p = 0.05)				

(a), (b), (c), (d) sorption/desorption parameters obtained by modelling with Langmuir model; (e) coefficient of multiple determination; (f) Scaled Root Mean Squared Error; (g) minimum error level of χ^2 test; (h) degrees of freedom = number of measurements – number of model parameters.

Table S2. Evaluated parameters with standard deviations and statistical indices for acetamiprid, imidacloprid and thiacloprid sorption/desorption equlibrium processes in the tested soils (S1 – S4) using Temkin nonlinear equilibrium model.

	Sorption				Desorption			
Fitted/statistical parameter	S1	S2	S3	S4	S1	S2	S3	S4
**Acetamiprid**								
K_T^{sor} (a, b) (L/mg)	1.36 (±0.03)	1.20 (±0.10)	1.11 (±0.11)	1.08 (±0.03)	2.52 (±1.61)	4.35 (±0.28)	4.60 (±0.45)	3.82 (±1.82)
B_1^{sor} (c, d) (mg/kg)	43.09 (±0.65)	16.23 (±0.21)	22.16 (±0.76)	26.09 (±0.12)	36.16 (±10.46)	8.68 (±0.19)	12.50 (±0.55)	18.12 (±3.09)
R^2 (e)	0.7614	0.7876	0.7365	0.7681	0.7334	0.7170	0.6684	0.7519
SRMSE (f)	0.4410	0.4413	0.4783	0.4084	0.3098	0.1725	0.2469	0.2932
err-% (g)	27.43	35.10	38.04	32.48	24.64	13.72	19.64	23.32
m (h)	4 (χ^2_{tab} = 9.488 at p = 0.05)							
**Imidacloprid**								
K_T^{sor} (a, b) (L/mg)	2.08 (±0.04)	1.19 (±0.13)	1.18 (±0.005)	1.64 (±0.12)	3.34 (±0.89)	6.58 (±5.46)	6.15 (±5.22)	7.05 (±5.56)
B_1^{sor} (c, d) (mg/kg)	54.25 (±0.87)	19.00 (±0.78)	23.99 (±0.08)	27.68 (±0.38)	45.42 (±1.21)	12.59 (±4.01)	17.70 (±6.40)	23.09 (±7.59)
R^2 (e)	0.7840	0.8840	0.8015	0.7676	0.7839	0.7584	0.6868	0.7523
SRMSE (f)	0.4008	0.2107	0.3636	0.4016	0.3155	0.2783	0.4214	0.4118
err-% (g)	31.88	16.75	28.91	31.93	25.09	22.13	33.51	32.75
m (h)	4 (χ^2_{tab} = 9.488 at p = 0.05)							
**Thiacloprid**								
K_T^{sor} (a, b) (L/mg)	3.66 (±1.33)	1.04 (±0.005)	1.34 (±0.22)	2.08 (±0.04)	13.88 (±0.35)	5.80 (±0.17)	11.62 (±0.15)	23.72 (±8.56)
B_1^{sor} (c, d) (mg/kg)	49.64 (±2.27)	29.94 (±0.28)	30.19 (±2.02)	31.02 (±2.80)	42.78 (±1.01)	19.96 (±0.19)	21.32 (±0.27)	22.31 (±2.13)
R^2 (e)	0.7665	0.9150	0.8249	0.7989	0.6549	0.7513	0.7199	0.6593
SRMSE (f)	0.4147	0.2198	0.3305	0.3628	0.5714	0.4558	0.6220	0.6975
err-% (g)	32.98	17.48	26.28	28.85	45.44	36.25	49.46	55.47
m (h)	4 (χ^2_{tab} = 9.488 at p = 0.05)							

(a), (b), (c), (d) sorption/desorption parameters obtained by modelling with Langmuir model; (e) coefficient of multiple determination; (f) Scaled Root Mean Squared Error; (g) minimum error level of χ^2 test; (h) degrees of freedom = number of measurements – number of model parameters.

Table S3. Matrix correlations analysis for soil properties and parameters obtained by Freundlich model for neonicotinoids sorption and desorption in the tested soils (S1 – S4) obtained by Principal Components Analysis (PCA). Bold typeface indicates statistically significant correlations at $p < 0.05$.

Variable	HA	pH	CEC	clay	TOC	Hum. acid	Ful. acid	Ratio 465/665	Ratio H/C	Ratio N/C	Ratio S/C	Ratio O/C	Ratio (N+O)/C
**HA**	1.00	**–0.71** ($p = 0.01$)	**0.98** ($p < 0.001$)	**–0.87** ($p < 0.001$)	**0.85** ($p < 0.001$)	–0.03	**0.91** ($p < 0.001$)	**0.84** ($p = 0.01$)	**–0.76** ($p = 0.004$)	**–0.99** ($p < 0.001$)	**–0.84** ($p = 0.001$)	**–0.77** ($p = 0.003$)	**–0.77** ($p = 0.003$)
**pH**	**–0.71** ($p = 0.01$)	1.00	**–0.82** ($p = 0.001$)	**0.93** ($p < 0.001$)	–0.34	–0.31	–0.38	–0.44	0.12	**0.62** ($p = 0.032$)	**0.98** ($p < 0.001$)	0.25	0.25
**CEC**	**0.98** ($p < 0.001$)	**–0.82** ($p = 0.001$)	1.00	**–0.91** ($p < 0.001$)	**0.80** ($p = 0.002$)	0.14	**0.81** ($p = 0.001$)	**0.82** ($p = 0.01$)	**–0.65** ($p = 0.021$)	**–0.94** ($p < 0.001$)	**–0.93** ($p < 0.001$)	**–0.72** ($p = 0.008$)	**–0.72** ($p = 0.008$)
**clay**	**–0.87** ($p < 0.001$)	**0.93** ($p < 0.001$)	**–0.91** ($p < 0.001$)	1.00	–0.49	0.02	**–0.67** ($p = 0.018$)	–0.52	–0.33	**0.83** ($p = 0.001$)	**0.95** ($p < 0.001$)	0.38	0.38
**TOC**	**0.85** ($p < 0.001$)	–0.34	**0.80** ($p = 0.002$)	–0.49	1.00	0.13	**0.85** ($p < 0.001$)	**0.97** ($p < 0.001$)	**–0.97** ($p < 0.001$)	**–0.86** ($p < 0.001$)	–0.53	**–0.99** ($p < 0.001$)	**–0.99** ($p < 0.001$)
**Hum. acid**	–0.03	–0.31	0.14	0.02	0.13	1.00	–0.35	0.32	0.04	0.15	–0.29	–0.21	–0.21
**Ful. acid**	**0.91** ($p < 0.001$)	–0.38	**0.81** ($p = 0.01$)	**–0.67** ($p = 0.018$)	**0.85** ($p < 0.001$)	–0.35	1.00	**0.76** ($p = 0.004$)	**–0.86** ($p < 0.001$)	**–0.96** ($p < 0.001$)	–0.55	**–0.78** ($p = 0.03$)	**–0.78** ($p = 0.003$)
**Ratio 465/665**	**0.84** ($p = 0.001$)	–0.44	**0.82** ($p = 0.001$)	–0.52	**0.98** ($p < 0.001$)	0.32	**0.76** ($p = 0.004$)	1.00	**–0.90** ($p < 0.001$)	**–0.82** ($p = 0.001$)	**–0.62** ($p = 0.033$)	**–0.98** ($p < 0.001$)	**–0.98** ($p < 0.001$)
**Ratio H/C**	**–0.76** ($p = 0.004$)	0.12	**–0.66** ($p = 0.021$)	0.33	**–0.97** ($p < 0.001$)	0.04	**–0.86** ($p < 0.001$)	**–0.91** ($p < 0.001$)	1.00	**0.79** ($p = 0.002$)	0.33	**0.97** ($p < 0.001$)	**0.97** ($p < 0.001$)
**Ratio N/C**	**–0.99** ($p < 0.001$)	**0.62** ($p = 0.032$)	**–0.94** ($p < 0.001$)	**0.83** ($p = 0.001$)	**–0.86** ($p = 0.001$)	0.15	**–0.96** ($p < 0.001$)	**–0.82** ($p = 0.001$)	**0.79** ($p = 0.002$)	1.00	**0.76** ($p = 0.004$)	**0.78** ($p = 0.003$)	**0.78** ($p < 0.001$)
**Ratio S/C**	**–0.84** ($p = 0.001$)	**0.98** ($p < 0.001$)	**–0.93** ($p < 0.001$)	**0.95** ($p < 0.001$)	–0.53	–0.29	–0.55	**–0.62** ($p = 0.033$)	0.33	**0.76** ($p = 0.004$)	1.000	0.45	0.45
**Ratio O/C**	**–0.77** ($p = 0.03$)	0.25	**–0.72** ($p = 0.003$)	0.38	**–0.99** ($p < 0.001$)	–0.21	**–0.78** ($p = 0.003$)	**–0.98** ($p < 0.001$)	**0.97** ($p < 0.001$)	**0.78** ($p = 0.003$)	0.45	1.00	**1.00** ($p < 0.001$)
**Ratio (N+O)/C**	**–0.77** ($p = 0.03$)	0.25	**–0.72** ($p < 0.001$)	0.38	**–0.99** ($p = 0.004$)	–0.21	**–0.78** ($p = 0.003$)	**–0.98** ($p < 0.001$)	**0.97** ($p < 0.001$)	**0.77** ($p = 0.001$)	0.45	**1.00** ($p < 0.001$)	1.00
K_F^{sor}	**0.78** ($p = 0.003$)	–0.52	**0.76** ($p = 0.004$)	**–0.64** ($p = 0.024$)	**0.71** ($p = 0.010$)	0.03	**0.72** ($p = 0.009$)	**0.70** ($p = 0.011$)	**–0.64** ($p = 0.025$)	**–0.77** ($p = 0.003$)	**–0.64** ($p = 0.026$)	**–0.66** ($p = 0.020$)	**–0.66** ($p = 0.020$)
K_{OC}	**0.64** ($p = 0.025$)	–0.49	**0.63** ($p = 0.027$)	**–0.59** ($p = 0.043$)	0.51	–0.03	0.57	0.50	–0.44	**–0.64** ($p = 0.028$)	–0.56	–0.45	–0.45
$1/n^{sor}$	0.44	**–0.68** ($p = 0.015$)	0.52	**–0.63** ($p = 0.027$)	0.15	0.16	0.23	0.22	–0.01	–0.39	**–0.65** ($p = 0.023$)	–0.09	–0.09
H	0.28	–0.39	0.29	–0.46	–0.02	–0.26	0.23	–0.04	0.07	–0.28	–0.35	0.10	0.10
ΔG^0	–0.56	0.50	–0.57	0.57	–0.40	0.01	–0.47	–0.41	0.32	0.55	0.55	0.34	0.34
λ	–0.26	0.36	–0.26	0.43	0.03	0.27	–0.21	0.05	–0.08	0.26	0.32	–0.11	–0.11
K_F^{des}	0.50	–0.23	0.47	–0.33	0.55	0.04	0.50	0.53	–0.52	–0.51	–0.33	–0.53	–0.53
$1/n^{des}$	0.53	**–0.65** ($p = 0.021$)	0.56	**–0.70** ($p = 0.011$)	0.19	–0.10	0.39	0.21	–0.08	–0.50	**–0.64** ($p = 0.026$)	–0.10	–0.10

[(a)] hydrolitic acidity; [(b)] cation exchange capacity; [(c)] total organic carbon; [(d)] carbon of humic acids; [(e)] carbon of fulvic acids; [(f), (g), (h), (i)] parameters obtained by modelling with Freundlich model; [(j)] organic carbon partition coefficient; [(k)] molar free Gibbs energy; [(l), (m)] hysteresis coefficients.

Table S4. Experimental conditions on the Atomic Absorption Spectrometer AAS 800 Perkin Elmer Analyst.

	Na^{+}	K^{+}	Ca^{2+}	Mg^{2+}
Concentration of standard (mg/L)	0.5; 1; 2; 3; 5	0.5; 1; 2;	0.5; 1; 2.5; 5; 10	0.5; 1; 2; 5
Wavelength (nm)	589.0	766.5	422.7	285.2
Crack opening (nm)	0.2	0.7	0.7	0.7
Measurement method	peak area	peak area	peak area	peak area
Acetylene flow (L/min)	2	2	2	2
Oxidant flow (L/min)	17	17	17	17

Table S5. Gradient program of mobile phases for chromatographic separation of insecticides.

Time (min)	Mobile phase A (%)	Mobile phase B (%)
00:00	98	2
01:00	98	2
15:00	2	98
18:00	2	98
18:05	98	2
20:00	98	2

Table S6. MS/MS conditions and MRM transitions of precursor-product ions of each insecticide studied and instrumental quality parameters.

Analyte	Q1 [(b)] (m/z)	Q3 [(b)] (m/z)	MRM Transition	CE [(c)] (V)	DP [(d)] (V)	EP [(e)] (V)	CEP [(f)] (V)	LOD[(g)] (ng/mL)	LOQ [(h)] (ng/mL)
Acetamiprid	223.2	126.1 [(a)]	Quantitation	39.0	50.0	10.0	10.0	0.030	< 0.1
	223.2	99.1	Confirmation	67.0	50.0	10.0	10.0		
Imidacloprid	256.2	209.0 [(a)]	Quantitation	44.0	50.0	10.0	10.0	0.028	< 0.1
	256.2	175.2	Confirmation	44.0	50.0	10.0	10.0		
Tiacloprid	253.1	126.1[(a)]	Quantitation	29.0	50.0	10.0	10.0	0.024	< 0.1
	253.1	99.1	Confirmation	57.0	50.0	10.0	10.0		

[(a)] transitions used in quantification; [(b)] quadrupole mass filter; [(c)] collision energy; [(d)] declustering potential; [(e)] exit potential; [(f)] voltage in the exit part of the collision cell; [(g)] limit of detection; [(h)] limit of quantitation.

Supplementary Materials References

1. Hinz, C. Description of Sorption Data with Isotherm Equations. *Geoderma* **2001**, *99*, 225–243, doi:10.1016/S0016-7061(00)00071-9.
2. Cox, L.; Koskinen, W.C.; Yen, P.Y. Sorption−Desorption of Imidacloprid and Its Metabolites in Soils. *J. Agric. Food Chem.* **1997**, *45*, 1468–1472, doi:10.1021/jf960514a.
3. Selim, H.M.; Zhu, H. Atrazine Sorption-Desorption Hysteresis by Sugarcane Mulch Residue. *J. Environ. Qual.* **2005**, *34*, 325–335.
